# Assessment of fish biodiversity in four Korean rivers using environmental DNA metabarcoding

**DOI:** 10.7717/peerj.9508

**Published:** 2020-07-14

**Authors:** Md Jobaidul Alam, Nack-Keun Kim, Sapto Andriyono, Hee-kyu Choi, Ji-Hyun Lee, Hyun-Woo Kim

**Affiliations:** 1 Interdisciplinary Program of Biomedical, Mechanical and Electrical Engineering, Pukyong National University, Busan, Republic of Korea; 2 Department of Marine, Fisheries and Marine Faculty, C Campus Jl. Mulyorejo Surabaya, Universitas Airlangga, Surabaya, East Java, Indonesia; 3 Molecular Ecology and Evolution Laboratory, Department of Biological Science, College of Science & Engineering, Sangji University, Wonju, Republic of Korea; 4 Department of Marine Biology, Pukyong National University, Busan, Republic of Korea

**Keywords:** Biodiversity, Korea, Next-generation sequencing, MiFish pipeline, eDNA metabarcoding

## Abstract

Environmental DNA (eDNA) metabarcoding is a cost-effective novel approach to estimate biodiversity in an ecosystem. In this study, the MiFish pipeline was employed to test if the system methodology is sufficiently reliable to estimate fish biodiversity in Korean rivers. A total of 125 unique haplotypes and 73 species were identified at the species level from 16 water samples collected from a single survey in four Korean rivers (Hyeongsan, Taehwa, Seomjin, and Nakdong). Among the four rivers, the highest species richness was recorded in the Seomjin River (52 species), followed by the Taehwa (42 species) and Hyeongsan (40 species) rivers. The Nakdong River (26 species) presented the lowest species richness and number of endemic species, presumably due to its metropolitan location and anthropogenic impacts, such as dams or weirs. We were also able to detect that five exotic species (*Carassius cuvieri, Cyprinus carpio, Cyprinus megalophthalmus, Lepomis macrochirus,* and *Micropterus salmoides*) are widely distributed in all surveyed rivers, a situation that might be problematic in terms of conservation. Our findings indicate that the eDNA metabarcoding technique is one of the most cost-effective scientific tools available for the management and conservation of the freshwater fish resources available in Korea. However, the low number of 12S sequences of endemic species in the database and low resolution of the MiFish region for differentiating several taxa should be upgraded for their wide use.

## Introduction

Fish communities have been considered as reliable bioindicators of ecosystem status due to their vulnerability to environmental or anthropogenic stresses such as pollution, climate change, or other disturbances in habitats (Dudgeon, 2010). Traditional monitoring methods for fish biodiversity, which have relied on the direct capture or observation of specimens, are often costly and time-consuming due to a lack of taxonomic expertise and the necessity of extensive fieldwork. Environmental DNA (eDNA) metabarcoding (detection of multispecies by using degraded DNA from environmental samples) has been proposed as an alternative strategy to analyze fish biodiversity, demonstrating the potential to improve the traditional methods in a cost-effective way (Foote et al., 2012; Kelly et al., 2017; Kelly et al., 2014; Shaw et al., 2016; Stoeckle, Soboleva & Charlop-Powers, 2017; Yamamoto et al., 2017). This technique has been shown to be sensitive as it allows the identification of rarely identified (Pilliod et al., 2013), invasive (Ardura et al., 2015; Cai et al., 2017; Clusa et al., 2017; Dejean et al., 2012; Klymus, Marshall & Stepien, 2017; Takahara, Minamoto & Doi, 2013; Williams et al., 2018), or migratory species (Gustavson et al., 2015; Pont et al., 2018; Yamamoto et al., 2016; Yamanaka & Minamoto, 2016).

Since eDNA metabarcoding analysis of fish biodiversity is mainly based on the amplicon of homologous genes by PCR, universal primers with high taxon-specificity and wide taxon-coverage are essential. Three fish-specific universal primer sets are currently reported: two sets for 12S rRNA regions (EcoPrimers (Riaz et al., 2011) and MiFish (Miya et al., 2015)) and one for the 16S rRNA region (Shaw et al., 2016). Among them, the MiFish primer set demonstrated reliability for eDNA metabarcoding analysis of fish biodiversity in both marine (Ushio et al., 2017; Yamamoto et al., 2017) and continental waters (Sato et al., 2018). More recently, the web-based MiFish pipeline in MitoFish was publicly open (http://mitofish.aori.u-tokyo.ac.jp/mifish/), alleviating the time-consuming bioinformatic analysis for the users (Sato et al., 2018).

Although metabarcoding analysis by the MiFish pipeline is one of the most reliable tools at the moment, numbers of MiFish sequences in the database are still one of the last hurdles to overcome for the global use of the MiFish pipeline. Since the average length of the MiFish region is approximately 170 bp, which is much smaller than the typically used 670 bp of the COI barcodes, a high-quality database is critical for successful species assignment. Species identification using the MiFish primer could not discriminate closely related species in several genera, including *Sebastes* spp. and *Takifugu* spp. (Yamamoto et al., 2017). In particular, considering the tremendous diversity of freshwater fishes, the direct application of the MiFish platform may produce a high amount of ‘unidentified’ records. In addition, a relatively much lower amount of MiFish sequence data (12S region) is currently deposited compared with those of the COI region. Therefore, before the direct application of the MiFish pipeline, the MiFish DNA sequence data for the local freshwater species should be tested for accurate fish biodiversity analysis using eDNA metabarcoding.

In this study, we first employed eDNA metabarcoding analysis of water samples collected from four rivers using the MiFish primer set in order to improve the knowledge regarding freshwater fish biodiversity in Korea. Next, we analyzed the haplotypes obtained by the MiFish pipeline to assess their compatibilities in the identification of endemic species of fishes inhabiting Korean rivers. We also calculated the Shannon-Wiener (*H*′) indices derived from the eDNA metabarcoding results to estimate fish biodiversity in four Korean rivers. Finally, the relationship between the fish assemblage according to the locations in the river was analyzed using heat-map clustering analysis.

## Materials and Methods

### Sample collection and environmental DNA extraction

**Figure 1:**
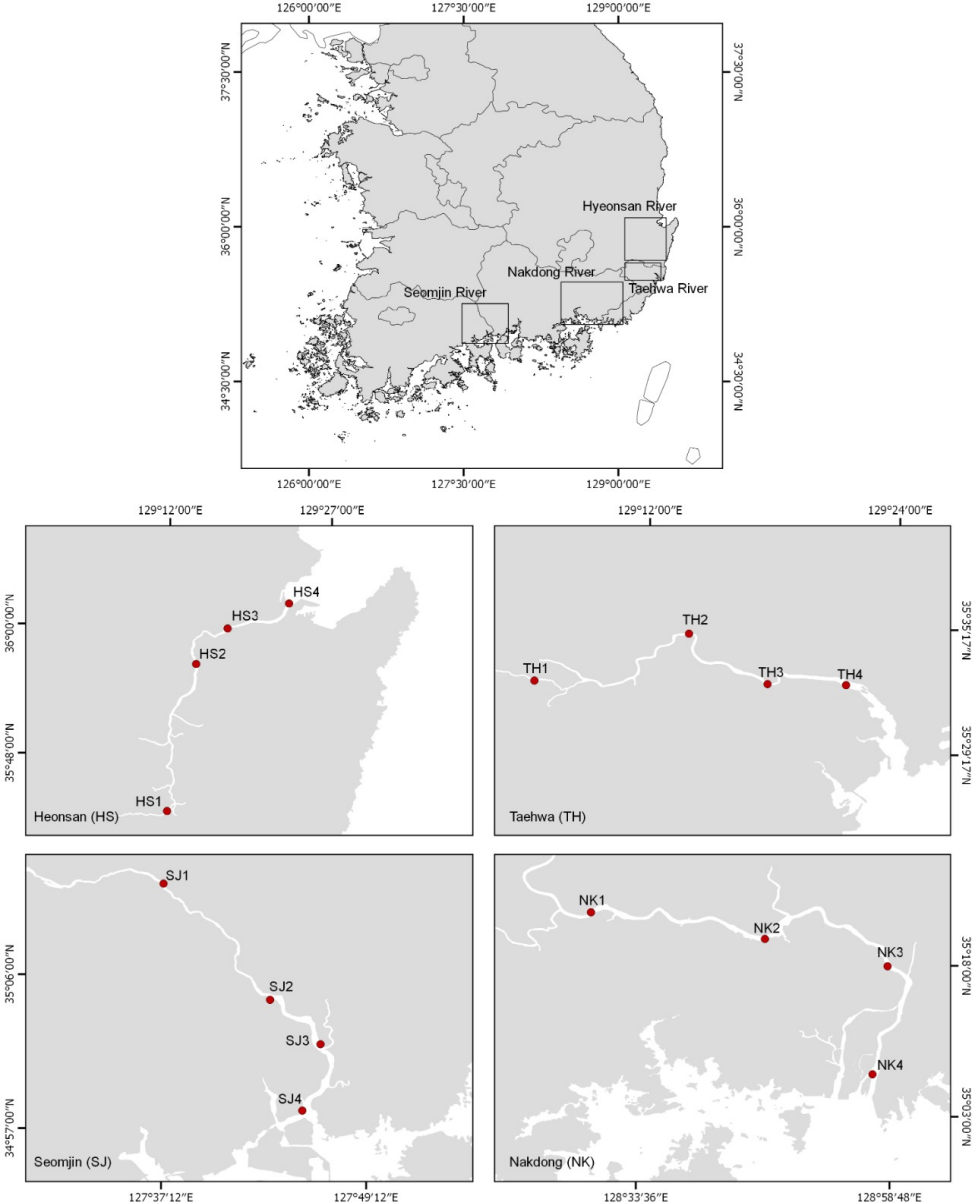
Water sample collection sites for environmental DNA metabarcoding study from four Korean rivers.

The eDNA water samples were collected on June 11 and 12, 2018 from 16 stations in the Hyeongsan, Taehwa, Seomjin, and Nakdong rivers, which are four large rivers in the southern part of the Korean Peninsula (Fig. 1 and Table 1). In this study, the sampling stations of each river were categorized as upstream (stations 1 and 2), midstream (station 3), and downstream (Station 4). One liter of water was collected at each station using disposable plastic bottles. After collecting the water, the bottles were immediately stored in an icebox and taken to the laboratory for filtration. Water temperature and salinity were measured with a conductivity meter (CD-4307SD, LUTRON). The water collected was filtered (250 mL × 4) with a 0.45 µm pore-sized GN-6 membrane (PALL Life Sciences, Mexico). The filtration system was cleaned with 10% commercial bleach containing sodium hypochlorite to prevent cross-contamination. After filtration, the membranes were put into 2.0 ml tubes and stored at −20 °C before DNA purification.

Genomic DNA was extracted directly from the membrane filters using the DNeasy® Blood and Tissue Kit (Qiagen, Germany), according to the manufacturer’s manual. The membrane filters were cut into smaller pieces before homogenization using a TissueLyser II motorized homogenizer (QIAGEN, Hilden, Germany). The extracted genomic DNA was quantified using a ND-1000 NanoDrop (Thermo Scientific, Waltham, MA, USA), aliquoted, and stored at −20 °C.

### Construction of the library and MiSeq sequencing

In order to assess the fish biodiversity, amplicon libraries of partial 12S rRNA region using the MiFish universal primer sets were constructed (Miya et al., 2015). The first PCR was performed to amplify the MiFish regions with an overhanging linker sequence for each Nextera XT index (Illumina, USA). The PCR mixture (20 µL) contained 1.0 µL of the MiFish (forward & reverse) primers (5pmol each), 2.0 µL template, 2.0 µL dNTPs (2.5 mM), 2.0 µL of 10X EX Taq buffer, 0.6 µL DMSO (3%), 0.2 µL of EXTaq Hot Start polymerase (TaKaRa Bio Inc. Japan), and 11.20 µL ultra-pure water. The PCR reaction began with denaturation at 95 °C for 3 min; followed by 30 cycles at 94 °C for 20 s, 65 °C for 15 s, and 72 °C for 15 s; and a final extension at 72 °C for 5 min. The amplicon with the expected size (250–350 bp) was purified with the AccuPrep® Gel Purification Kit (Bioneer, Republic of Korea) after 1.5% agarose gel electrophoresis. The purified amplicons were subjected to additional PCR to link each amplicon with the corresponding Nextera XT index. The second PCR mixture (20 µL) contained 5 µL template, 1 µL of a couple of index primers (10 pmol), 0.5 µL dNTPs (10 mM), 4 µL 5X Phusion HF Buffer, 8.3 µL ultrapure water, and 0.2 µL Phusion Hot Start Flex DNA polymerase (New England Biolabs, Hitchen, UK). The second PCR started at 94 °C for 5 min; followed by 15 cycles at 94 °C for 30 s, 55 °C for 30 s, and 72 °C for 30 s; and an additional 5 min at 72 °C. No noticeable bands were detected in the desired ranges for 16 field negative controls in 1.5% agarose gel electrophoresis. Consequently, the 16 negative controls were discarded from the following analyses. After gel purification, the quality and quantity of the indexed PCR products with the expected sizes were analyzed using the Qubit dsDNAHS Assay Kit (Invitrogen, Carlsbad, CA, USA), followed by sequencing using the MiSeq platform (2 × 300 bp).

**Table 1:** Environmental DNA sample collection sites with physico-chemical parameters of the four Korean rivers.

River	Date	Station	GPS location	Temp. (°C)	Salinity (PSU)
Hyeongsan	2018.06.11	HS1	N35°42′36″, E129°11′42″	18.6	1.00
		HS2	N35°56′14″, E129°14′24″	19.5	2.02
		HS3	N35°59′32″, E129°17′19″	20.0	3.20
		HS4	N36°01′51″, E129°23′01″	24.0	20.20
Taehwa	2018.06.11	TH1	N35°32′52″, E129°06′27″	19.4	1.02
		TH2	N35°35′07″, E129°13′52″	19.8	2.04
		TH3	N35°32′42″, E129°17′38″	22.7	14.02
		TH4	N35°32′39″, E129°21′24″	19.2	17.80
Seomjin	2018.06.12	SJ1	N35°11′18″, E127°37′21″	24.2	0.15
		SJ2	N35°04′30″, E127°43′35″	23.4	2.01
		SJ3	N35°01′54″, E127°46′32″	23.0	12.9
		SJ4	N34°58′01″, E127°45′28″	23.25	16.8
Nakdong	2018.06.12	ND1	N35°23′19″, E128°29′09″	24.0	1.92
		ND2	N35°20′40″, E128°46′26″	24.1	2.40
		ND3	N35°17′57″, E128°58′37″	23.2	2.78
		ND4	N35°07′13″, E128°57′07″	22.5	4.50

### Bioinformatic analysis of the NGS data

The MiSeq raw reads were paired using Python 2.7 (Zhang, 2015), and the paired reads were uploaded to the MiFish pipeline (http://mitofish.aori.u-tokyo.ac.jp/mifish/) for further analyses. In the MiFish pipeline, a low-quality tail of reads (QV ≤ 20) was trimmed in FASTQC. After taxonomic assignments from the MiFish pipeline, the sequences assigned to OTUs were compared with the GenBank database. If the sequence identity of the query sequence and top BLASTN hit was ≥99%, the sequence was ascertained as a particular species. If the sequence identity ranged from 97% to 99%, the sequence was ascertained to the genus level, whereas sequences ranging from 97% to 95% were assigned as ‘unidentified’ genera. The geographic distribution of each species was assessed on the FishBase website (http://www.fishbase.org/search.php). Alpha biodiversity was measured using the normalized read numbers from each sampling station of the four rivers sampled. The Shannon-Wiener (*H*′) index indicates the heterogeneity of species or the richness of species in an ecosystem (Gray, 2000; Magurran, 1988). The *H*′ index and the heat map clustering analysis were calculated using the PRIMER® v7 software (Clarke & Gorley, 2015).

## Results

### Physicochemical parameters

The water temperature of the sample sites ranged from 18.6 °C to 24.20 °C (Table 1). The Hyeongsan River showed the highest temperature difference (5.4 °C) between upstream (HS1) to downstream (HS4), whereas the lowest levels of temperature variation were observed in the Seomjin (0.8 °C) and Nakdong (1.5 °C) rivers. The lowest salinity (0.15 PSU) was measured at station 1 (upstream) of the Seomjin River, while the highest (20.20 PSU) was recorded at station 4 (downstream) of the Hyeongsan River. The salinity level increased from upstream to downstream in all rivers, except in the Nakdong River, where an artificial dam was constructed to block water from the ocean (Table 1).

### Analysis of fish haplotypes obtained using the MiFish pipeline

**Figure 2:**
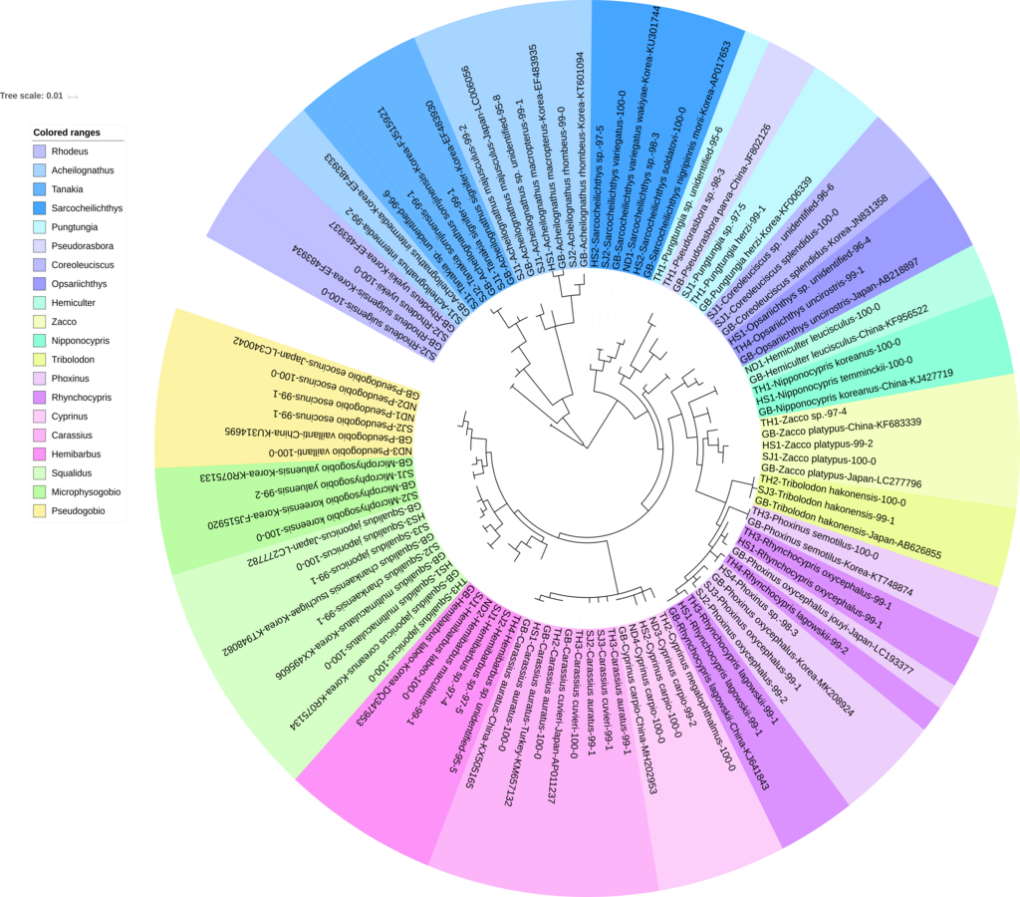
Phylogenetic tree analysis of fish species under the family Cyprinidae detected from four Korean rivers. Phylogenetic tree was constructed by Maximum likelihood (ML) algorithm (MEGA 7.0) under the 1000 replication bootstrap.

**Figure 3:**
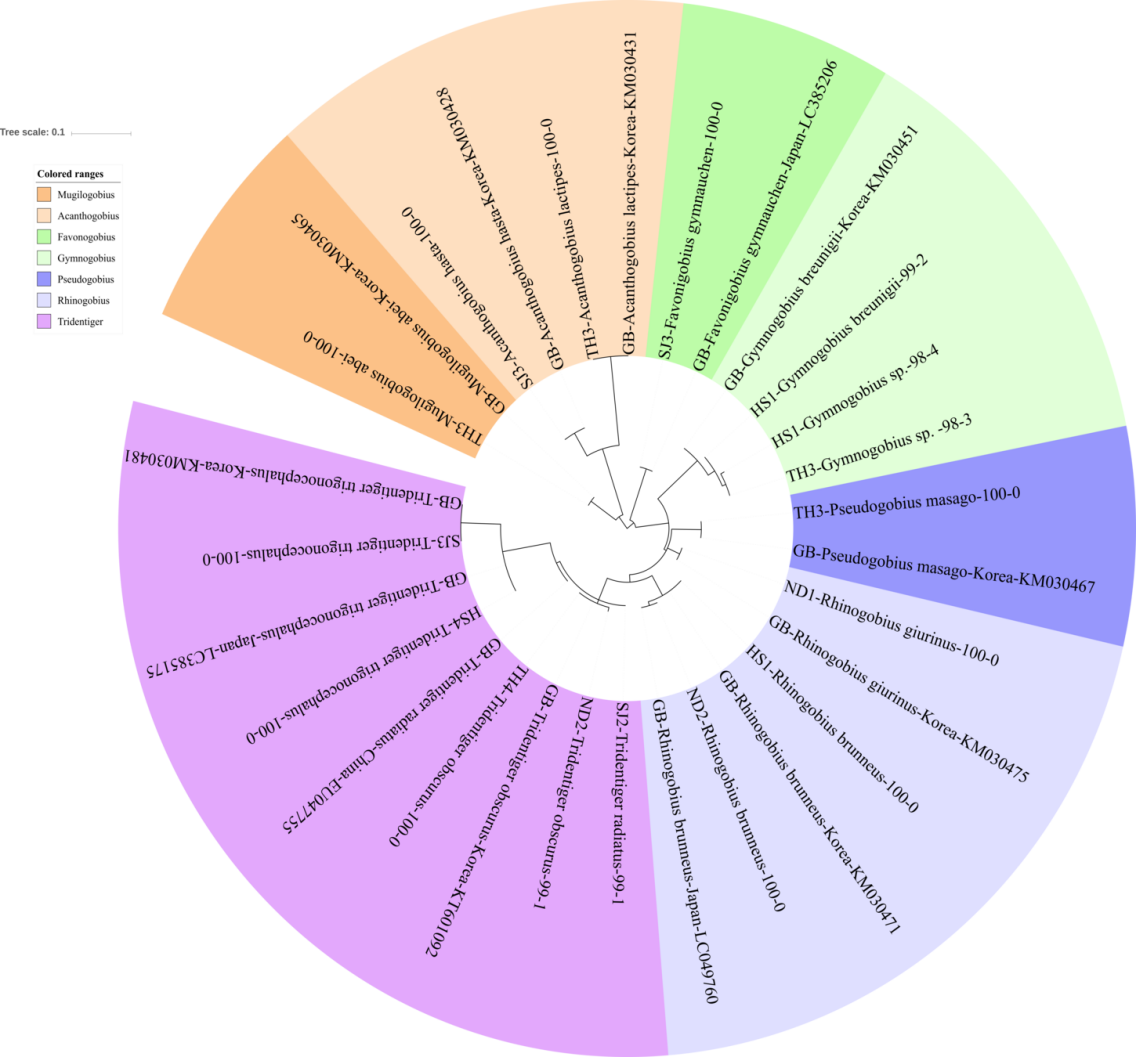
Phylogenetic tree analysis of fish species under the family Gobiidae. Phylogenetic tree was constructed by Maximum likelihood (ML) algorithm (MEGA 7.0) under the 1000 replication bootstrap.

**Figure 4:**
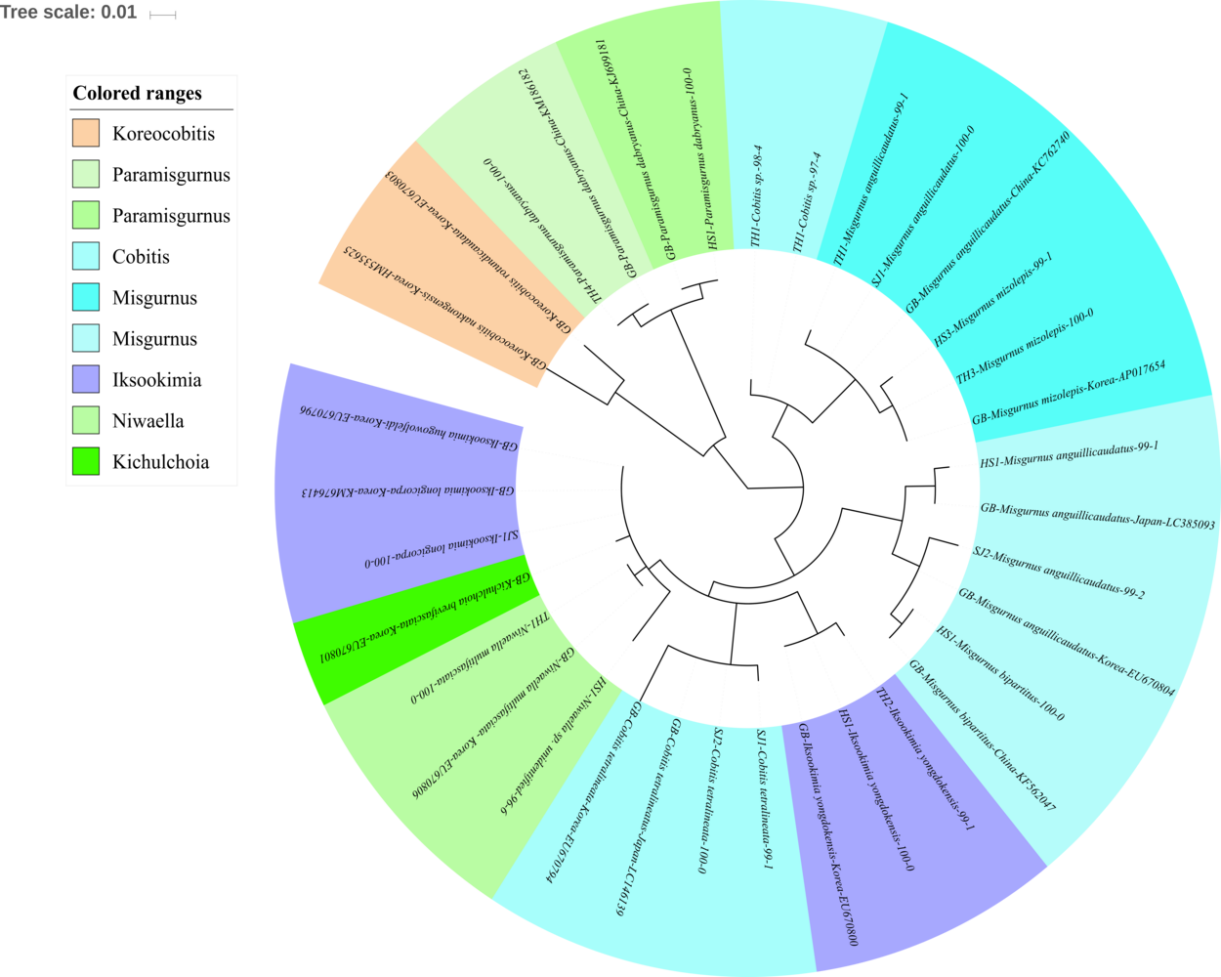
Phylogenetic tree analysis of fish species under the family Cobitidae. Phylogenetic tree was constructed by Maximum likelihood (ML) algorithm (MEGA 7.0) under the 1000 replication bootstrap.

**Figure 5:**
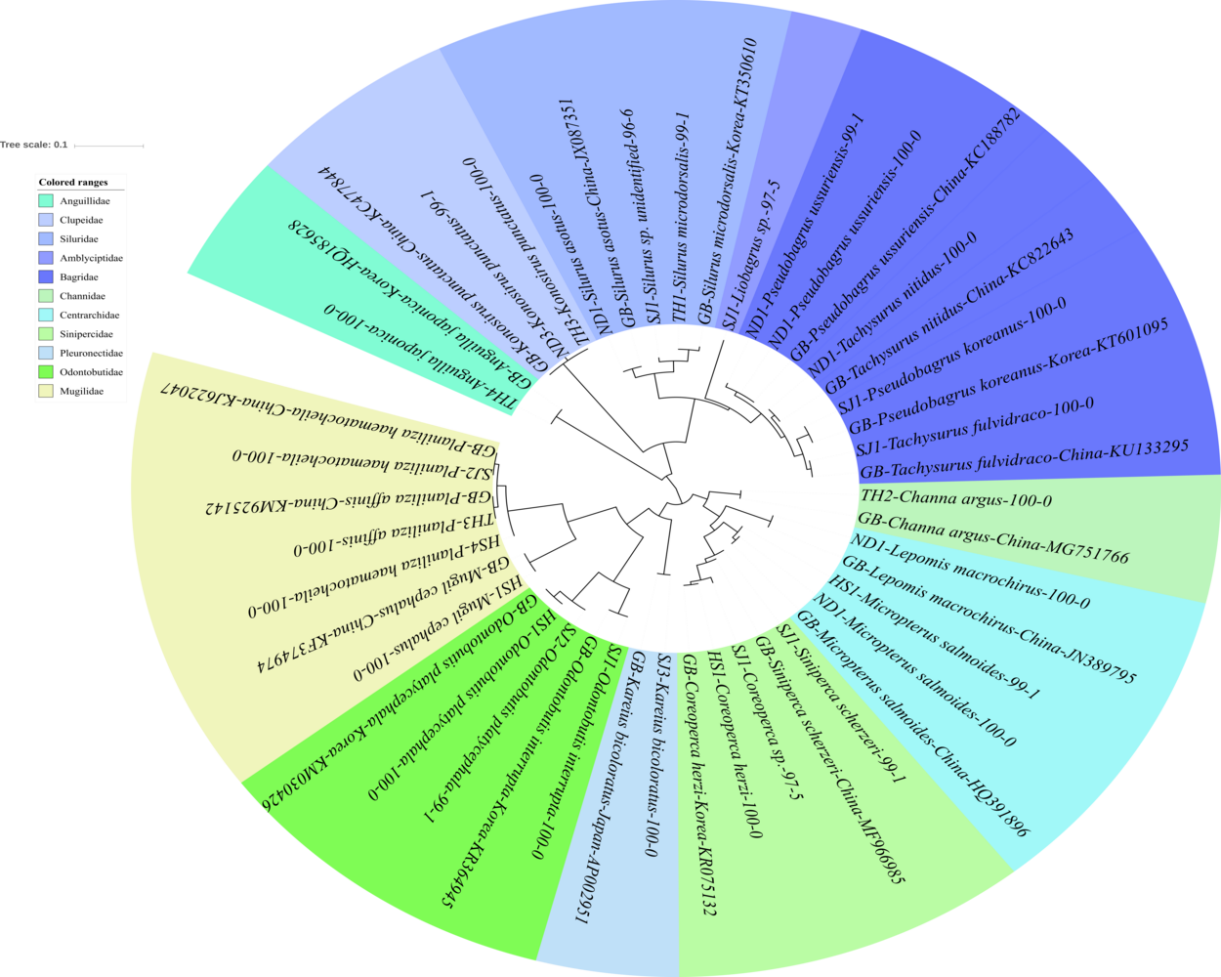
Phylogenetic tree analysis of fish species under the other families of Teleostei. Phylogenetic tree was constructed by Maximum likelihood (ML) algorithm (MEGA 7.0) under the 1000 replication bootstrap.

The reliability of the MiFish pipeline (http://mitofish.aori.u-tokyo.ac.jp/mifish/workflows/new) for the biodiversity assessment of fish species inhabiting the sampled rivers was analyzed (Table 2). From 2,315,605 raw reads, 2,280,850 merged reads were obtained by the MiFish pipeline, with a 98.50% yield from the raw reads. A total of 238 representative haplotypes were assigned to the default cutoff sequence identity. Among the 238 haplotypes, 125 unique haplotypes were identified using the phylogenetic tree analysis in the MEGA 7 software (Kumar, Stecher & Tamura, 2016) with a maximum likelihood algorithm (Figs. 2–5). A total of 2,241,130 reads (98.26%) were assigned to 73 confirmed species, 46 genera, and 13 families of Teleostei, with 99% as cutoff identity. The remaining 39,720 reads (49 haplotypes), which showed less than 99% identity, were further assigned to11 genera and eight unidentified genera (Table 3). A total of 34,755 reads (1.50%) were discarded from further analyses. The highest species number was identified in the family Cyprinidae (35), followed by Gobiidae (11), and Cobitidae (8), while the remaining (19) were from other families of Teleostei. Among them, the highest species number (4 species) was identified in the genus *Acheilognathus*, followed by *Carassius, Misgurnus, Squalidus,* and *Tridentiger* with three species in each of those genera (Table S1).

**Table 2:** Summary of taxonomic assignment of the MiSeq reads from four Korean rivers.

	Seomjin River	Taehwa River	Hyeongsan River	Nakdong River	Total
Raw reads	561,473	609,755	601,165	543,212	2,315,605
Processed merged reads	553,175	600,744	592,281	534,650	2,280,850
Total haplotypes	76	67	53	42	238 (125)^a^
Haplotypes with species name	61	49	48	31	189 (105)^a^
Total species	52	42	40	26	160 (73)^a^

**Notes.**

a Final number, after removal of duplicated one in brackets.

**Table 3:** List of fish haplotypes with the GenBank numbers identified from the eDNA metabarcoding study of the four rivers.

No.	Family	Haplotype ID	Haplotypes	Identity (%)	Korean haplotype	Chinese haplotype	Japanese haplotype	Others
1	Gobiidae	SJ3	*Acanthogobius hasta*	100	KM030428	KM891736	–	
2	Gobiidae	TH3	*Acanthogobius lactipes*	100	KM030431	–	LC385140	
3	Cyprinidae	SJ1	*Acheilognathus intermedia*	99	EF483933	–	–	
4	Cyprinidae	HS1	*Acheilognathus macropterus*	99	EF483935	KJ499466	LC092100	
5	Cyprinidae	SJ1	*Acheilognathus majusculus*	99	–	–	LC006056	
6	Cyprinidae	SJ2	*Acheilognathus rhombeus*	99	KT601094	–	LC146100	
7	Cyprinidae	SJ1	*Acheilognathus* sp. (unidentified)	95			LC006056	
8	Anguillidae	TH4	*Anguilla japonica*	100	HQ185628	MH050933	LC193417	
9	Cyprinidae	HS1	*Carassius auratus*	100	–	KX505165		
10	Cyprinidae	TH2	*Carassius auratus*	100				Turkey KM657132
11	Cyprinidae	TH3	*Carassius auratus*	99		AY771781	LC193299	
12	Cyprinidae	SJ2	*Carassius auratus*	99	–	AY771781	LC193299	
13	Cyprinidae	TH3	*Carassius cuvieri*	100	–	–	AP011237	
14	Cyprinidae	SJ3	*Carassius cuvieri*	100			AP011237	
15	Channidae	TH1	*Channa argus*	100	–	MG751766	AB972107	
16	Cobitidae	TH1	*Cobitis* sp.	97	EU670794	–	LC146139	
17	Cobitidae	TH1	*Cobitis* sp.	97	EU670794	–	LC146139	
18	Cobitidae	SJ2	*Cobitis tetralineata*	100	EU670794	–	LC146139	
19	Cobitidae	SJ1	*Cobitis tetralineata*	99	EU670794	–	LC146139	
20	Cyprinidae	SJ1	*Coreoleuciscus* sp. (unidentified)	96	JN831358	–	AP011258	
21	Cyprinidae	SJ1	*Coreoleuciscus splendidus*	100	JN831358	–	AP011258	
22	Sinipercidae	HS3	*Coreoperca herzi*	100	KR075132	–	–	
23	Sinipercidae	SJ1	*Coreoperca* sp.	97	KR075132	–	–	
24	Cyprinidae	ND4	*Cyprinus carpio*	100	–	KX710076	AP017363	
25	Cyprinidae	HS2	*Cyprinus carpio*	100	–	KX710076	AP017363	
26	Cyprinidae	ND3	*Cyprinus carpio*	99	–	KX710076	AP017363	
27	Cyprinidae	TH2	*Cyprinus megalophthalmus*	100	–	KR869143	–	
28	Gobiidae	SJ3	*Favonigobius gymnauchen*	100	–	–	LC385206	
29	Gobiidae	HS1	*Gymnogobius breunigii*	99	KM030451	–	–	
30	Gobiidae	HS1	*Gymnogobius* sp.	98	KM030451	–	–	
31	Gobiidae	TH3	*Gymnogobius* sp.	98	KM030451	–	–	
32	Cyprinidae	SJ1	*Hemibarbus labeo*	100	DQ347953	KP064328	LC049898	
33	Cyprinidae	ND2	*Hemibarbus maculatus*	99	–	NC018534		
34	Cyprinidae	SJ1	*Hemibarbus* sp.	97	DQ347953	KP064328	LC049898	
35	Cyprinidae	SJ2	*Hemibarbus* sp.	97	DQ347953	KP064328	LC049898	
36	Cyprinidae	TH4	*Hemibarbus* sp. (unidentified)	95	DQ347953	KP064328	LC049898	
37	Cyprinidae	ND1	*Hemiculter leucisculus*	100	–	–	LC340359	
38	Cobitidae	SJ1	*Iksookimia longicorpa*	100	KM676413	–	LC146135	
39	Cobitidae	HS1	*Iksookimia yongdokensis*	100	EU670800	–	–	
40	Cobitidae	TH2	*Iksookimia yongdokensis*	99	EU670800	–	–	
41	Pleuronectidae	SJ3	*Kareius bicoloratus*	100	–	–	AP002951	
42	Clupeidae	TH3	*Konosirus punctatus*	100	–	KC477844	LC020951	Taiwan AP011612
43	Clupeidae	ND3	*Konosirus punctatus*	99	–	KC477844	LC020951	Taiwan AP011612
44	Centrarchidae	TH4	*Lepomis macrochirus*	100	–	JN389795	AP005993	USA KP013118
45	Amblycipitidae	SJ1	*Liobagrus* sp.	97	KR075136	KX096605	AP012015	
46	Cyprinidae	SJ2	*Microphysogobio koreensis*	100	FJ515920	–	–	
47	Cyprinidae	SJ1	*Microphysogobio yaluensis*	99	KR075133	–	AP012073	
48	Centrarchidae	ND1	*Micropterus salmoides*	100	–	HQ391896	LC069536	USA DQ536425
49	Centrarchidae	HS1	*Micropterus salmoides*	99	–	HQ391896	LC069536	USA DQ536425
50	Cobitidae	SJ1	*Misgurnus anguillicaudatus*	100	–	KC762740	–	
51	Cobitidae	TH1	*Misgurnus anguillicaudatus*	99	–	KC762740	–	
52	Cobitidae	SJ2	*Misgurnus anguillicaudatus*	99	EU670804	–	–	
53	Cobitidae	HS1	*Misgurnus anguillicaudatus*	99	–	–	LC385093	
54	Cobitidae	HS1	*Misgurnus bipartitus*	100	–	KF562047	LC091592	
55	Cobitidae	TH3	*Misgurnus mizolepis*	100	AP017654	–	–	
56	Cobitidae	HS3	*Misgurnus mizolepis*	99	AP017654	–	–	
57	Mugilidae	HS1	*Mugil cephalus*	100	–	KF374974	LC278014	
58	Gobiidae	TH3	*Mugilogobius abei*	100	KM030465	–	LC421743	Taiwan KF128984
59	Cyprinidae	TH1	*Nipponocypris koreanus*	100	–	KJ427719	–	
60	Cyprinidae	HS1	*Nipponocypris temminckii*	100	–	–	AP012116	
61	Cobitidae	TH1	*Niwaella multifasciata*	100	EU670807	–	LC146133	
62	Cobitidae	HS1	*Niwaella* sp. (unidentified)	96	EU670807	–	LC146133	
63	Odontobutidae	SJ1	*Odontobutis interrupta*	100	KR364945	–	–	
64	Odontobutidae	HS1	*Odontobutis platycephala*	100	KM030426	–	–	
65	Odontobutidae	SJ2	*Odontobutis platycephala*	99	KM030426			
66	Cyprinidae	HS1	*Opsariichthys* sp. (unidentified)	96	–	–	AB218897	
67	Cyprinidae	TH3	*Opsariichthys uncirostris*	99	–	–	AB218897	
68	Cobitidae	TH4	*Paramisgurnus dabryanus*	100	–	KM186182	LC146125	
69	Cobitidae	HS1	*Paramisgurnus dabryanus*	100	–	KJ699181	LC146125	
70	Cyprinidae	SJ2	*Phoxinus oxycephalus*	99	MK208924	–	AB626852	
71	Cyprinidae	SJ3	*Phoxinus oxycephalus*	99	MK208924	–	AB626852	
72	Cyprinidae	TH3	*Phoxinus semotilus*	100	KT748874	–	–	
73	Mugilidae	TH3	*Planiliza affinis*	100	–	KM925142	LC277843	
74	Mugilidae	SJ2	*Planiliza haematocheila*	100	–	KJ622047	LC021099	
75	Mugilidae	HS4	*Planiliza haematocheila*	100	–	KJ622047	LC021099	
76	Bagridae	SJ1	*Pseudobagrus koreanus*	100	KT601095	–	–	
77	Bagridae	ND1	*Pseudobagrus ussuriensis*	100	–	KC188782	–	
78	Bagridae	ND2	*Pseudobagrus ussuriensis*	99	–	KC188782	–	
79	Cyprinidae	ND2	*Pseudogobio esocinus*	100	–	–	LC340042	
80	Cyprinidae	ND1	*Pseudogobio esocinus*	99	–	–	LC340042	
81	Cyprinidae	ND3	*Pseudogobio vaillanti*	100	–	KU314695	LC146041	
82	Cyprinidae	SJ2	*Pseudogobio vaillanti*	99	–	KU314695	LC146041	
83	Gobiidae	TH3	*Pseudogobius masago*	100	KM030467	–	LC049791	
84	Cyprinidae	TH1	*Pungtungia herzi*	99	KF006339	–	AB239598	
85	Cyprinidae	SJ1	*Pungtungia* sp.	97	KF006339	–	AB239598	
86	Cyprinidae	TH1	*Pungtungia* sp. (unidentified)	96	KF006339	–	AB239598	
87	Gobiidae	HS1	*Rhinogobius brunneus*	100	KT601096	–		
88	Gobiidae	ND2	*Rhinogobius brunneus*	100			LC049760	
89	Gobiidae	ND1	*Rhinogobius giurinus*	100	KM030475	KP892753	LC049748	
90	Cyprinidae	SJ2	*Rhodeus suigensis*	100	EF483934	–	–	
91	Cyprinidae	SJ1	*Rhodeus uyekii*	100	EF483937	–	–	
92	Cyprinidae	HS1	*Rhynchocypris lagowskii*	99	–	KJ641843	–	
93	Cyprinidae	TH3	*Rhynchocypris lagowskii*	99		KJ641843		
94	Cyprinidae	TH4	*Rhynchocypris lagowskii*	99		KJ641843		
95	Cyprinidae	SJ2	*Rhynchocypris oxycephalus*	99	–	–	LC193377	
96	Cyprinidae	SJ3	*Rhynchocypris oxycephalus*	99			LC193377	
97	Cyprinidae	HS4	*Rhynchocypris* sp.	98			LC193377	
98	Cyprinidae	HS2	*Sarcocheilichthys soldatovi*	100	–	–	LC146036	
99	Cyprinidae	HS2	*Sarcocheilichthys* sp.	97	KU301744	–	AP012067	
100	Cyprinidae	ND3	*Sarcocheilichthys* sp.	97	KU301744	–	AP012067	
101	Cyprinidae	SJ2	*Sarcocheilichthys variegatus*	100	KU301744	–	AP012067	
102	Siluridae	ND1	*Silurus asotus*	100	–	JX087351	NC015806	
103	Siluridae	TH1	*Silurus microdorsalis*	99	KT350610	–	–	
104	Siluridae	SJ1	*Silurus* sp. (unidentified)	96	KT350610			
105	Sinipercidae	SJ1	*Siniperca scherzeri*	100	–	MF966985	–	Taiwan AP014527
106	Cyprinidae	SJ2	*Squalidus chankaensis*	100	KT948082	–	–	
107	Cyprinidae	HS3	*Squalidus japonicus*	100			LC277782	
108	Cyprinidae	SJ3	*Squalidus japonicus*	99			LC277782	
109	Cyprinidae	TH3	*Squalidus japonicus coreanus*	100	KR075134	–		
110	Cyprinidae	HS1	*Squalidus multimaculatus*	100	KX495606	–	–	
111	Bagridae	SJ1	*Tachysurus fulvidraco*	100	–	KU133295	LC193372	
112	Bagridae	ND2	*Tachysurus nitidus*	100	–	KC822643	–	
113	Cyprinidae	SJ1	*Tanakia signifer*	99	EF483930	–	–	
114	Cyprinidae	SJ2	*Tanakia somjinensis*	99	FJ515921	–	–	
115	Cyprinidae	SJ1	*Tanakia* sp.(unidentified)	96	FJ515921			
116	Cyprinidae	TH2	*Tribolodon hakonensis*	100	–	–	AB626855	
117	Cyprinidae	SJ3	*Tribolodon hakonensis*	99	–	–	AB626855	
118	Gobiidae	TH4	*Tridentiger obscurus*	100	KT601092	MF663787	LC193168	
119	Gobiidae	SJ2	*Tridentiger radiatus*	99	–	EU047755	–	
120	Gobiidae	ND2	*Tridentiger radiatus*	99				
121	Gobiidae	SJ3	*Tridentiger trigonocephalus*	100	KM030481			
122	Gobiidae	HS4	*Tridentiger trigonocephalus*	100		KT282115	LC385175	
123	Cyprinidae	SJ1	*Zacco platypus*	100	–		LC277796	
124	Cyprinidae	HS1	*Zacco platypus*	99		KF683339		
125	Cyprinidae	TH1	*Zacco* sp.	97		KF683339		

### Cyprinidae

A total of 65 haplotypes were identified in the family Cyprinidae. Among the 65 haplotypes, 51 were assigned to 35 species of fishes with ≥ 99% of sequence identity to the GenBank database (Fig. 2). Two haplotypes in the genus *Hemibarbus* from the Seomjin River (SJ1) and the Nakdong River (ND2) showed 100% and 99% identity to the sequences of *Hemibarbus labeo* (GenBank Number: DQ347953) and *Hemibarbus maculatus* (LC146032) sampled in Korea and Japan, respectively. Among the four endemic species in the genus *Hemibarbus*, *H. labeo* and *H. longirostris* are the most widely distributed species in Korea (Lee et al., 2012). Two haplotypes identified from the Seomjin River (SJ1 and SJ2) and one from Taehwa River (TH1) showed 97% and 95% identity to a sequence of *H. longirostris* (LC049889), respectively, which suggests that these three haplotypes may be either *H. longirostris* or *H. mylodon* (Fig. 2).

Five haplotypes were identified in the genus *Squalidus*. Four species of the genus have been reported from Korean waters: *Squalidus gracilis*, *S. japonicus, S. multimaculatus, and S. chankaensis* (Kim & Park, 2002). Two haplotypes from the Taehwa (TH3) and Hyeongsan rivers *(HS1) showed 100% identity to sequences of S. japonicas coreanus* (GenBank Number: KR075134) and *S. multimaculatus* (GenBank Number: KT948081), respectively. *Another haplotype from the* Hyeongsan River *(HS3) showed 100% identity to a sequence of S. japonicas* (GenBank Number: LC277782) sampled in Japan. Two haplotypes from the Seomjin River showed 99% identity to a sequence of *S. chankaensis tsuchigae* (GenBank Number: KT948082) sampled in Korea.

Fishes of the subfamily Acheilognathinae, commonly known as bitterlings, deposit eggs in the gill cavities of freshwater mussels (Kitamura, 2007; Kitamura et al., 2012). Approximately 60 species of bitterlings are considered valid in the genera *Acheilognathus*, *Tanakia*, and *Rhodeus* (Arai, 1988). *Acheilognathus intermedia, A. macropterus, A. majusculus, A. rhombeus, Rhodeus suigensis, R. uyekii, Tanakia somjinensis,* and *T. signifier* were identified with a sequence identity >99% when compared to the GenBank database*.* Three haplotypes from the Seomjin River showed 99% sequence identity to the respective haplotypes of *A. intermedia* (EF483933), *T. somjinensis* (FJ515921), and *T. signifier* (EF483930) sampled in Korea. Among them, *T. somjinensis* and *T. signifier* are endemic to Korea (Kim & Park, 2002). One haplotype from the Taehwa River (TH3) showed 100% identity to a sequence of *Rhynchocypris semotilus* (KT748874) sampled in Korea. This species is currently categorized as Critically Endangered in the Red Data Book of endangered fishes in Korea (Ko, Kim & Park, 2011).

Two sub-species of *Sarcocheilichthys* are known in Korea: *S. nigripinnis morii* and *S. variegates wakiyae* (Kim & Park, 2002). Two haplotypes from the Seomjin (SJ2) and Hyeongsan (HS2) rivers showed 100% and 97%, respectively, identity to a sequence of *S. variegatus wakiyae* (GenBank Number: KU301744) sampled in Korea. One haplotype from the Hyeongsan River (HS2) showed 100% and 99.43% identity to a sequence of *S. soldatovi* (LC146036) and the Korean haplotype of *S. nigripinnis morii* (AP017653) sampled in Japan and Korea, respectively. However, *S. soldatovi* is not currently reported for Korean waters. Therefore, further studies are needed to confirm the occurrence of this species in the Hyeongsan River for conservation purposes.

### Gobiidae

We identified 16 haplotypes of the family Gobiidae, representing seven genera and 11 species (Fig. 3). Five haplotypes were identified in the genus *Tridentiger*, which represents the five known species of the genus recorded in Korea (Kim et al., 2005). One haplotype from the Taehwa River (TH4) showed 100% identity with a sequence of *T. obscures* (GenBank Number: KT601092) sampled in Korea. One haplotype from the Hyeongsan River (HS4) showed 100% identity to a sequence of *T. trigonocephalus* (GenBank Number: LC385175) sampled in Japan, and another haplotype from the Seomjin River (SJ3) showed 100% identity to a sequence of *T. trigonocephalus* (GenBank Number: KM030481) sampled in Korea. According to the recovered phylogenetic tree, the *T. trigonocephalus* haplotype from the Seomjin River is different from that of the Hyeongsan River (Fig. 3). All three haplotypes of the genus *Rhinogobius* showed 100% identity to the database. The first and second haplotypes showed 100% identity to sequences of *R. brunneus* sampled in Korea (KM030471) and Japan (LC049760), respectively. The third haplotype showed 100% identity to a sequence of *R. giurinus* sampled in Korea (KM030475). Two haplotypes of *Gymnogobius* sp. from the Taehwa and Hyeongsan rivers showed 98% sequence identity to *G. taranetzi* (GenBank Number: LC385155). Nine species of the genus *Gymnogobius* are currently reported in Korea (Kim et al., 2005), and their MiFish sequences should be supplemented to the GenBank database.

### Cobitidae

Sixteen species in five genera of the family Cobitidae are currently reported from Korean rivers (Kim, 2009). A total of 18 haplotypes, representing five genera of the family, were identified (Fig. 4). Two haplotypes in the genus *Cobitis* identified in the Seomjin River were most closely related to *C. tetralineata* (LC146139) sampled in Japan, with 100% and 99% sequence identity. Two haplotypes from the Taehwa River showed 98% and 97% identity to *C. hankugensis* (LC146140). Two species of *Misgurnus* are reported from the Korean waters, *M. mizolepis* and *M. anguillicaudatus* (Kim, 2009). Interestingly, two phylogenetically distinct clades in *M. anguillicaudatus* were identified in the phylogenetic analysis (Fig. 4). One of them was grouped with the haplotype of *M. bipartitus* (KF562047) sampled in China, while the other was clustered with the *M. mizolepis* (AP017654) sampled in Korea. *Misgurnus bipartitus* is currently reported to be endemic to China, and sequence data of Korean freshwater fishes in GenBank data should be reexamined.

Two haplotypes from the Hyeongsan River (HS1; KJ699181) and the Taehwa River (TH4; KM186182) showed 100% identity with haplotypes of *Paramisgurnus dabryanus* sampled in China (Fig. 4). This species is regarded as endemic to China, but *P. dabryanus* is often imported to Korea together with *Misgurnus anguillicaudatus* due to their phenotypic similarity. Shimizu & Takagi (2010) concluded that there are different populations of *P. dabryanus*, and the two haplotypes of the species identified herein suggest that *P. dabryanus* has been imported from various locations in China. One *haplotype* from the Taehwa River (TH1) showed 100% identity to a sequence of *Niwaella multifaciata* (EU670806) sampled in Korea, while another from the Hyeongsan River (HS1) showed a lower (96%) identity to *Niwaella* sp. Therefore, further studies should be conducted to confirm the presence of species of this genus in the Hyeongsan River.

### Other families of Teleostei

In addition to the three main families of Teleostei identified in this study, 27 additional haplotypes were found in the samples. These haplotypes represented 19 species belonging to14 genera and 11 families, namely Amblycipitidae (1), Anguillidae (1), Bagridae (5 haplotypes), Centrarchidae (3), Channidae (1), Clupeidae (2), Mugilidae (4), Odontobutidae (3), Pleuronectidae (1), Siluridae (3), and Sinipercidae (3). All the haplotypes of the family Bagridae were clearly identified and included: *Pseudobargrus ussuriensis*, *P. koreanus*, *Tachysurrus nitidus*, and *T. fulvidraco* (Fig. 5). Two species of *Silurus* are currently known in Korean rivers, *S. microdorsalis* and *S. asotus* (Park & Kim, 1994). One haplotype from the Taehwa River (TH1) showed 99% identity to a sequence of *Silurus microdorsalis* (GenBank Number: KT350610) sampled in Korea, whereas another haplotype from the Seomjin River (SJ1) showed a lower identity (96%) with *S. microdorsalis* (KT350610) sampled in Korea.

One haplotype of the Amblycipitidae from the Seomjin River showed 97% and 96% identity to *Liobagrus styani* (KX096605) and *L. mediadiposalis* (KR075136), sampled in China and Korea, respectively. These results indicate that haplotypes of the family should be supplemented for accurate identification. Three species of *Odontobutis* are currently known in Korea: *O. interrupta*, *O. platycephala*, and *O. obscura* (Kim et al., 2005). Two of them (*O. interrupta* and *O. platycephala*) were identified in this study with 100% identity to the sequences of *O. interrupta* and *O. platycephala* sampled in Korea (KR364945 and KM030426). Two haplotypes of the genus *Coreoperca* showed 100% and 97% sequence identity to *Coreoperca herzi* (KR075132) sampled in Korea*.* Since two species of *Coreoperca* are reported to be endemic to the Korean Peninsula (Kim et al., 2005), the second haplotype is most likely *C. kawamebari*, but further studies should be conducted to confirm this identification. Two invasive species of the family Centrarchidae, the Bluegill (*Lepomis macrochirus*) and the Largemouth bass (*Micropterus salmoides*) were also identified in this study. These two species are endemic to North America but were introduced in the Korean Peninsula for aquaculture purposes without considering their impact on local ecosystems.

### Fish biodiversity in the four rivers

**Figure 6:**
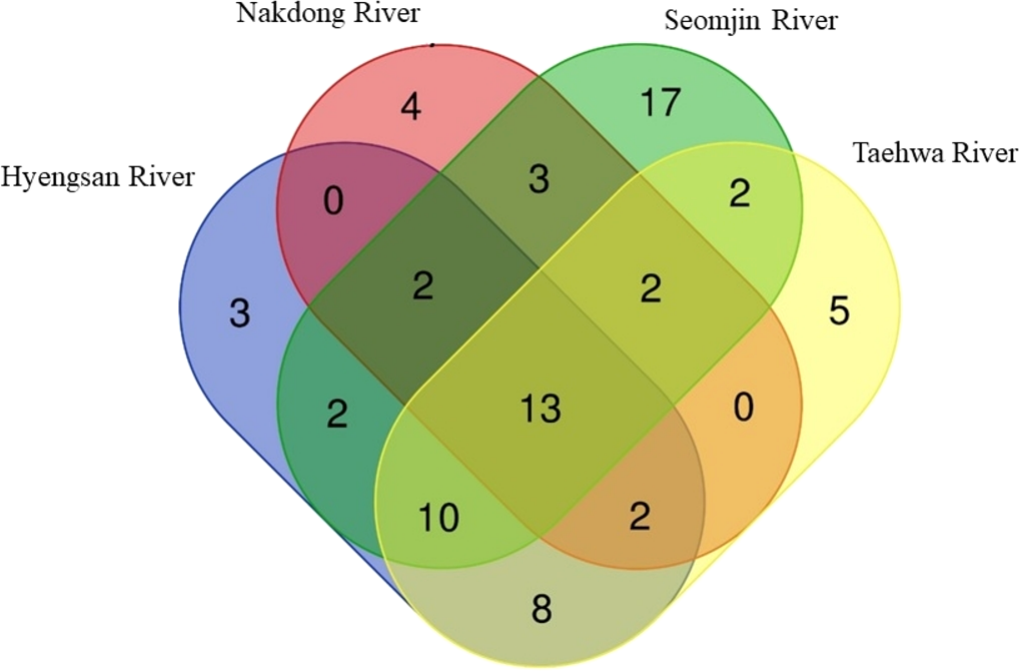
Venn diagram of identified species of fishes in the four Korean rivers. Venn diagram was constructed with an online program (http://bioinformatics.psb.ugent.be/webtools/Venn/).

**Figure 7:**
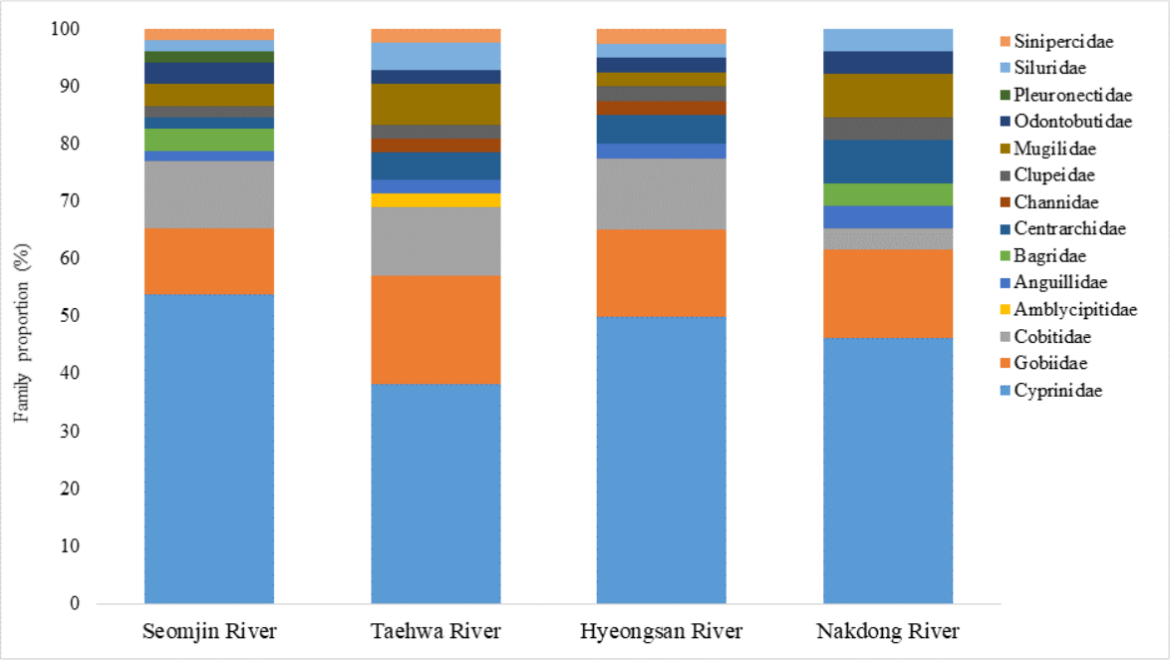
Proportion of families detected from the four Korean rivers by environmental DNA metabarcoding.

Fish assemblages in the four rivers included in this study were analyzed. Among the 73 confirmed fish species detected in this study, 13 were identified in all four rivers: *Anguilla japonica, Hemibarbus labeo, Konosirus punctatus, Micropterus salmoides, Misgurnus mizolepis, Mugil cephalus, Opsariichthys uncirostris, Pseudorasbora parva, Rhinogobius brunneus, Rhynchocypris lagowskii, Silurus asotus, Tridentiger obscurus,* and *Zacco platypus* (Fig. 6). Regardless of sample stations, species of the Cyprinidae appear to be dominant, with average proportions of 47.02 ± 6.73%, followed by the Gobiidae (15.24 ± 3.07%) and Cobitidae (9.95 ± 4.09%; Fig. 7). However, the proportions of species in those families were different between upstream and downstream stations. The proportion of Cyprinidae species was higher (45.27 ± 9.1%) upstream (stations 1 and 2) than downstream (33.78 ± 18% at station 4). In contrast, the proportion of Gobiidae was lower (14.53 ± 8.28%) upstream than downstream (station 4, 19.90 ± 14%).

The highest number of species was recorded in the Seomjin River (52 species), followed by the Taehwa (42 species), Hyeongsan (40 species), and Nakdong (26 species) rivers. A total of 17 species were exclusively recorded in the Seomjin River: *Acanthogobius hasta, Acheilognathus intermedia, A. majusculus, A. rhombeus, Cobitis tetralineata, Coreoleuciscus splendidus, Kareius bicoloratus, Microphysogobio yaluensis, Phoxinus oxycephalus, Pseudobagrus koreanus, Rhodeus suigensis, R. uyekii, Sarcocheilichthys variegatus, Siniperca scherzeri, Squalidus gracilis, Tanakia somjinensis,* and *T. signifier.* Five species were only recorded in the Taehwa River: *Acanthogobius lactipes,Mugilogobius abei, Pseudogobius masago, Rhynchocypris semotilus,* and *Silurus microdorsalis*, whereas four species were only identified in the Nakdong River: *Plagiognathops microlepis*, *Pseudobagrus ussuriensis, Rhinogobius giurinus,* and *Tachysurus nitidus.* Finally, only three species (*Nipponocypris koreanus, Sarcocheilichthys soldatovi,* and *Squalidus multimaculatus*) were exclusively recorded in the Hyeongsan River (Fig. 6).

The highest Shannon index (SI) was identified in the Seomjin River (3.480), followed by the Taehwa (3.067), Hyeongsan (2.954), and Nakdong (2.864) rivers. Among the 16 surveyed stations, station 1 of the Seomjin River (SJ1) showed the highest species richness (2.197), whereas the lowest richness (1.008) was recorded atthe station 4 of the Nakdong River (ND4). From upstream to downstream, average species richness decreased from 1.951 to 1.415 (Table 4).

**Table 4:** Shannon Index (SI) measured from four Korean rivers by eDNA metabarcoding.

	Seomjin River	Taehwa River	Hyeongsan River	Nakdong River	Average
Station 1	2.197	2.073	1.755	1.777	1.951
Station 2	2.182	1.941	1.709	1.734	1.892
Station 3	2.125	1.631	1.691	1.465	1.728
Station 4	2.105	1.443	1.102	1.008	1.415
Overall SI index	3.48	3.067	2.954	2.864	–

### Clustering analysis

**Figure 8:**
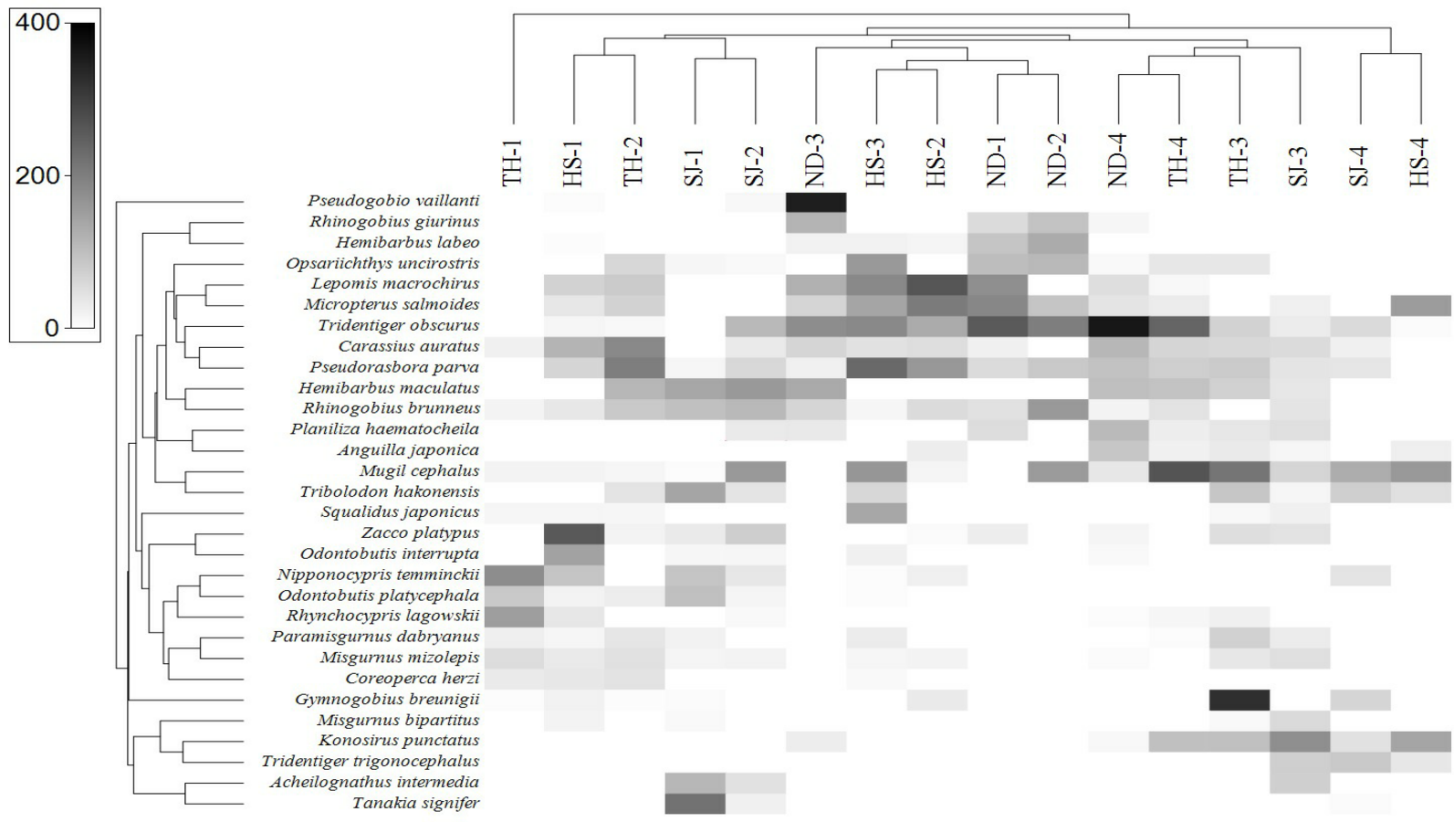
Heat map analysis of top 30 fish species identified in 16 sampling stations of the four Korean rivers. Heat map analysis was constructed by Primer v7 program.

In order to assess the correlation between the fish assemblage and sample stations, we conducted a heat-map analysis with the 30 most abundant species using Primer software (Clarke & Gorley, 2015). The results indicate the species distribution in different sampling stations (Fig. 8). In upstream sites (Stations 1 and 2), the dominant species were *A. intermedia, Coreoperca herzi, Misgurnus mizolepis, Nipponocypris temminckii, Rhynchocypris lagowskii, Odontobutis interrupta, O. platycephala, Tanakia signifier,* and *Zacco platypus*. At station 3, the dominant species were *Gymnogobius breunigii, Mugil cephalus, Pseudorasbora parva, Rhinogobius giurinus,* and *R. brunneus*. Finally, in the downstream sample (Station 4), *Anguilla japonica, Konosirus punctatus, Mugil cephalus, Planiliza haematocheila, Tridentiger obscurus,* and *T. trigonocephalus* were identified as the dominant species, all of which were either euryhaline or anadromous (https://www.fishbase.org).

## Discussion

The results indicate that eDNA metabarcoding using the MiFish pipeline is a useful tool for assessing fish biodiversity in Korean freshwater ecosystems, since a total of 125 unique haplotypes, including at least 73 species, were successfully identified by a single-day survey of 16 sampling stations in four rivers (Figs. 2– 5). According to the “Survey and Evaluation of Aquatic Ecosystem Health (SEAEH)”, a total of 130 freshwater fish species were identified from 953 sampling sites that covered most of the Korean rivers and lakes (Yoon et al., 2012). The total number of species confirmed by eDNA metabarcoding was equivalent to approximately 56% of those obtained by the year-long conventional surveys. The efficiency of eDNA metabarcoding might actually be even higher, especially considering the number of haplotypes successfully identified at the genus and/or family level. This result indicates that eDNA metabarcoding with the MiFish pipeline can significantly contribute to the assessment of freshwater fish biodiversity in Korea, especially considering its relatively lower cost of implementation when compared with more conventional morphology-based surveys. Although the methodology in each research group may be slightly different, similar conclusions have been reached in other studies (Bista et al., 2017; Deiner et al., 2016). eDNA metabarcoding analysis is also adequate for surveying aquatic species in protected areas, as it minimizes disturbance of vulnerable communities (Fernandez et al., 2018).

Despite its relevance as a methodology for the assessment of biodiversity, there are still a few shortcomings for a more widespread use of eDNA metabarcoding by the MiFish pipeline. First, MiFish sequence data for endemic species of Korea should be supplemented to the GenBank database. According to the Archive of Korean species (https://species.nibr.go.kr), 67 species of freshwater fishes are endemic to Korea, and many of their MiFish sequences are still not available in the GenBank database. In addition to the lack of sequence data, freshwater fishes typically have intra-species genetic distances that are generally higher than those of marine species (Seehausen & Wagner, 2014). Second, the MiFish primer amplifies the 12S rRNA gene (163–185 bp) region of mitochondrial DNA, which is smaller and less variable than the COI region, which is typically used in species identification (Ivanova et al., 2007). In fact, the MiFish region was unable to differentiate several closely related marine fish taxa, such as those in the genus *Sebastes* and *Takifugu* (Sato et al., 2018; Yamamoto et al., 2017). We also found that the average genetic distance of several genera in the family Cyprinidae was low in the MiFish region. For example, the average genetic distance of *Carassius* species was too low (0.01) and the identification at the species level was not possible (Fig. 2).

Further studies using eDNA metabarcoding might also be relevant to obtain more than biodiversity data, such as the quantitative analysis of fish species. It is difficult to estimate the spatial abundance of eDNA in lotic environments. In fact, many factors should be considered for the quantitative analysis of eDNAs in rivers, including water dynamics (Deiner & Altermatt, 2014; Jerde et al., 2016; Wilcox et al., 2016) or different decaying times due to different physical, chemical, or biological factors (Shapiro, 2008). It is generally known that shorter fragments of DNA are degraded slower than larger ones, increasing their probability of detection in natural environments (Deagle, Eveson & Jarman, 2006). Therefore, it is still too early to adopt eDNA metabarcoding for the quantitative analysis of fish species under natural conditions. For the quantitative study, standardized collection methods and pretreatment procedures for NGS sequencing analysis should also be established. One of the strongest points in the biodiversity survey by eDNA metabarcoding is the quantity of information it can generate compared with more conventional surveys since large datasets are useful for statistical analyses. However, large amounts of data have been produced using different water collection methods, eDNA preparation, sequencing, and bioinformatic analysis platforms by different research groups in different countries. Therefore, the interconversion of data is currently not possible. The establishment of an international standard regarding the overall methodology of eDNA metabarcoding would help researchers to produce more comparable data.

According to the results obtained in this study, the highest species richness was found in the Seomjin River (3.48) compared with those of the other three rivers: the Taehwa River (3.06), Hyeongsan River (2.95), and Nakdong River (2.86). The lower values of species richness detected in the Nakdong, Hyeongsan, and Taehwa rivers are presumably related to the higher anthropogenic alteration of the natural conditions in those rivers. Like most other Korean rivers, these three rivers run through highly populated metropolitan cities, in which rivers are exposed to various human impacts that directly or indirectly promote changes in the diversity and distribution of freshwater fishes (Finkenbine, Atwater & Mavinic, 2000). In particular, the lowest species richness (2.86) and number of endemic species (only one, *Odontobutis interrupta*) were identified in the Nakdong River, where the highest number of constructions and population exist among the sampled rivers. Lee et al. (2015) reported only two endemic species (*Coreoperca herzi* and *Odontobutis platycephala*) in the Nakdong River using a conventional catch survey. Moreover, eight endemic species (*Coreoleuciscus splendidus, Iksookimia longicorpa, Microphysogobio koreensis, M. yaluensis, Odontobutis interrupta, O. platycephala, Pseudobagrus koreanus,* and *Squalidus gracilis*) were identified in this study in the Seomjin River, a number that is similar to those obtained in previous studies (Jang, Lucas & Joo, 2003; Lee et al., 2015). Several constructions along urbanized watersheds, including dams and weirs, have caused the simplification and reduction of habitats, decreasing the biodiversity in the river (Nilsson et al., 2005; Riley et al., 2005). In contrast, there is no metropolitan city along the Seomjin River, which is, therefore, less exposed to anthropogenic impacts. A long-term survey should be conducted to establish a clear correlation between anthropogenic factors and fish assemblages in the Korean rivers.

The eDNA metabarcoding analysis also indicates that some exotic fish species are widely distributed in Korean rivers. We were able to identify at least five exotic fish species: *Carassius cuvieri, Cyprinus carpio, C. megalophthalmus, Lepomis macrochirus*, and *Micropterus salmoides* (Table S3). These exotic species may affect native fishes in terms of shelter and spawning sites. They can also disturb the food chain, preying on native fish. In addition, these species have a high reproductive capacity, which makes them important potentially invasive species (Keller & Lake, 2007;Koster, 2002). Surprisingly, our results also revealed that the largemouth bass, *M. salmoides*, and the bluegill, *L. macrochirus*, are likely present in all the sampled rivers. These two species, which are native to North America, were artificially introduced in the 1970s in Korea as freshwater fish stock, without any further consideration of the effects on the freshwater ecosystems of the country. They are now widely distributed throughout the Korean Peninsula, competing with the native species. A long-term survey of these rivers should be conducted to properly assess the potential impacts of these introduced species (Jang et al., 2002; Yoon et al., 2012). Freshwater ecosystems are much more vulnerable to invasive species, causing biodiversity loss and global climate change (Clavero & García-Berthou, 2005), and eDNA metabarcoding analyses would be useful for monitoring the distribution patterns of invasive species in Korean rivers.

##  Supplemental Information

10.7717/peerj.9508/supp-1 Supplemental Information 1 Track changed manuscript file from Professional English language service

10.7717/peerj.9508/supp-2 Table S1 List of fish species identified from the eDNA metabarcoding study from four Korean rivers

10.7717/peerj.9508/supp-3 Table S2 Genetic distance of species under the family Cyprinidae

10.7717/peerj.9508/supp-4 Table S3 List of exotic species identified by eDNA metabarcoding study from four Korean rivers

## Additional Information and Declarations

### Data Availability

The following information was supplied regarding data availability: The sequences are available at NCBI Bio Project (BioProject ID: PRJNA630155; Submission ID, SUB7373098).
SAMN14826804, SAMN14826805, SAMN14826806, SAMN14826807, SAMN14826808, SAMN14826809, SAMN14826810, SAMN14826811, SAMN14826812, SAMN14826813, SAMN14826814, SAMN14826815, SAMN14826816, SAMN14826817, SAMN14826818, SAMN14826819.
