# Supplementary material for: Assessment of fish biodiversity in four Korean rivers using environmental DNA metabarcoding
Source: PeerJ. 2020 Jul 14;8:e9508. doi: 10.7717/peerj.9508 (PMC7367050; doi:10.7717/peerj.9508)
Supplement: Supplemental Information 1 [file peerj-08-9508-s001.docx]

**Assessment of fish biodiversity in four Korean rivers using environmental DNA metabarcoding**

Md. Jobaidul Alam^1^, Nack-Keun Kim^1^, Sapto Andriyono^1,2^, Hee-kyu Choi^3^, Ji-Hyun Lee^4^, and Hyun-Woo Kim^1,4^*

^1^Interdisciplinary Program of Biomedical, Mechanical and Electrical Engineering, Pukyong National University, Busan, 48513, Republic of Korea

^2^Fisheries and Marine Faculty, C Campus Jl. Mulyorejo Surabaya 60115. Universitas Airlangga, Surabaya, East Java, Indonesia

^3^Molecular Ecology and Evolution Laboratory, Department of Biological Science, College of Science & Engineering, Sangji University, Wonju 26339, Republic of Korea

^4^Department of Marine Biology, Pukyong National University, Busan 48513, Republic of

Korea

* Corresponding author:

Hyun-Woo Kim, Ph. D

Department of Marine Biology

Pukyong National University

48513, Republic of Korea

Tel: 82-51-629-5926

Fax: 82-51-629-5930

E-mail: kimhw@pknu.ac.kr

**ABSTRACT**

Environmental DNA (eDNA) metabarcoding is a cost-effective novel approach to estimate biodiversity in an ecosystem. In this study, the MiFish pipeline was employed to test if the system methodology is sufficiently reliable to estimate fish biodiversity in Korean rivers. A total of 125 unique haplotypes and 73 species were identified at the species level from 16 water samples collected from a single survey in four Korean rivers (Hyeongsan, Taehwa, Seomjin, and Nakdong). Among the four rivers, the highest species richness was recorded in the Seomjin River (52 species), followed by the Taehwa (42 species) and Hyeongsan (40 species) rivers. The Nakdong River (26 species) presented the lowest species richness and number of endemic species, presumably due to its metropolitan location and anthropogenic impacts, such as dams or weirs. We were also able to detect that five exotic species (*Carassius cuvieri, Cyprinus carpio, Cyprinus megalophthalmus, Lepomis macrochirus,* and *Micropterus salmoides*) are widely distributed in all surveyed rivers, a situation that might be problematic in terms of conservation. Our findings indicate that the eDNA metabarcoding technique is one of the most cost-effective scientific tools available for the management and conservation of the freshwater fish resources available in Korea. However, the low number of 12S sequences of endemic species in the database and low resolution of the MiFish region for differentiating several taxa should be upgraded for their wide use.

Keywords: biodiversity, Korea, next-generation sequencing, MiFish, metabarcoding, eDNA

**INTRODUCTION**

Fish communities have been considered as reliable bioindicators of ecosystem status due to their vulnerability to environmental or anthropogenic stresses such as pollution, climate change, or other disturbances in habitats ([Dudgeon, 2010](#_ENREF_12)). Traditional monitoring methods for fish biodiversity, which have relied on the direct capture or observation of specimens, are often costly and time-consuming due to a lack of taxonomic expertise and the necessity of extensive fieldwork. Environmental DNA (eDNA) metabarcoding (detection of multispecies by using degraded DNA from environmental samples) has been proposed as an alternative strategy to analyze fish biodiversity, demonstrating the potential to improve the traditional methods in a cost-effective way ([Foote et al., 2012](#_ENREF_15); [Kelly et al., 2017](#_ENREF_22); [Kelly et al., 2014](#_ENREF_23); [Shaw et al., 2016](#_ENREF_46); [Stoeckle et al., 2017](#_ENREF_48); [Yamamoto et al., 2017](#_ENREF_53)). This technique has been shown to be sensitive as it allows the identification of rarely identified ([Pilliod et al., 2013](#_ENREF_39)), invasive ([Ardura et al., 2015](#_ENREF_2); [Cai et al., 2017](#_ENREF_4); [Clusa et al., 2017](#_ENREF_7); [Dejean et al., 2012](#_ENREF_11); [Klymus et al., 2017](#_ENREF_29); [Takahara et al., 2013](#_ENREF_49); [Williams et al., 2018](#_ENREF_52)), or migratory species ([Gustavson et al., 2015](#_ENREF_17); [Pont et al., 2018](#_ENREF_40); [Yamamoto et al., 2016](#_ENREF_54); [Yamanaka and Minamoto, 2016](#_ENREF_55)).

Since eDNA metabarcoding analysis of fish biodiversity is mainly based on the amplicon of homologous genes by PCR, universal primers with high taxon-specificity and wide taxon-coverage are essential. Three fish-specific universal primer sets are currently reported: two sets for 12S rRNA regions [Eco Primers ([Riaz et al., 2011](#_ENREF_41)) and MiFish ([Miya et al., 2015b](#_ENREF_36))] and one for the 16S rRNA region ([Shaw et al., 2016](#_ENREF_46)). Among them, the MiFish primer set demonstrated reliability for eDNA metabarcoding analysis of fish biodiversity in both marine ([Ushio et al., 2017](#_ENREF_50); [Yamamoto et al., 2017](#_ENREF_53)) and continental waters ([Sato et al., 2018](#_ENREF_43)). More recently, the web-based MiFish pipeline in MitoFish was publicly open (<http://mitofish.aori.u-tokyo.ac.jp/mifish/>), alleviating the time-consuming bioinformatic analysis for the users ([Sato et al., 2018](#_ENREF_43)).

Although metabarcoding analysis by the MiFish pipeline is one of the most reliable tools at the moment, numbers of MiFish sequences in the database are still one of the last hurdles to overcome for the global use of the MiFish pipeline. Since the average length of the MiFish region is approximately 170 bp, which is much smaller than the typically used 670 bp of the COI barcodes, a high-quality database is critical for successful species assignment. Species identification using the MiFish primer could not discriminate closely related species in several genera, including *Sebastes* spp. and *Takifugu* spp. ([Yamamoto et al., 2017](#_ENREF_53)). In particular, considering the tremendous diversity of freshwater fishes, the direct application of the MiFish platform may produce a high amount of ‘unidentified’ records. In addition, a relatively much lower amount of MiFish sequence data (12S region) is currently deposited compared with those of the COI region. Therefore, before the direct application of the MiFish pipeline, the MiFish DNA sequence data for the local freshwater species should be tested for accurate fish biodiversity analysis using eDNA metabarcoding.

In this study, we first employed eDNA metabarcoding analysis of water samples collected from four rivers using the MiFish primer set in order to improve the knowledge regarding freshwater fish biodiversity in Korea. Next, we analyzed the haplotypes obtained by the MiFish pipeline to assess their compatibilities in the identification of endemic species of fishes inhabiting Korean rivers. We also calculated the Shannon-Wiener (H') indices derived from the eDNA metabarcoding results to estimate fish biodiversity in four Korean rivers. Finally, the relationship between the fish assemblage according to the locations in the river was analyzed using heat-map clustering analysis.

**MATERIALS AND METHODS**

**Sample collection and environmental DNA extraction**

The eDNA water samples were collected on June 11 and 12, 2018 from 16 stations in the Hyeongsan, Taehwa, Seomjin, and Nakdong rivers, which are four large rivers in the southern part of the Korean Peninsula (Fig.1 and Table 1). In this study, the sampling stations of each river were categorized as upstream (stations 1 and 2), midstream (station 3), and downstream (Station 4). One liter of water was collected at each station using disposable plastic bottles. After collecting the water, the bottles were immediately stored in an icebox and taken to the laboratory for filtration. Water temperature and salinity were measured with a conductivity meter (CD-4307SD, LUTRON). The water collected was filtered (250 mL × 4) with a 0.45 µm pore-sized GN-6 membrane (PALL Life Sciences, Mexico). The filtration system was cleaned with 10 % commercial bleach containing sodium hypochlorite to prevent cross-contamination. After filtration, the membranes were put into 2.0 ml tubes and stored at -20 °C before DNA purification.

Genomic DNA was extracted directly from the membrane filters using the DNeasy® Blood and Tissue Kit (Qiagen, Germany), according to the manufacturer’ s manual. The membrane filters were cut into smaller pieces before homogenization using a TissueLyser II motorized homogenizer (QIAGEN, Hilden, Germany). The extracted genomic DNA was quantified using a ND-1000 NanoDrop (Thermo Scientific, Waltham, MA, USA), aliquoted, and stored at -20 °C.

**Construction of the library and MiSeq sequencing**

In order to assess the fish biodiversity, amplicon libraries of partial 12S rRNA region using the MiFish universal primer sets were constructed ([Miya et al., 2015a](#_ENREF_35)). The first PCR was performed to amplify the MiFish regions with an overhanging linker sequence for each Nextera XT index (Illumina, USA). The PCR mixture (20 µL) contained 1.0 µL of the MiFish (forward & reverse) primers (5pmol each), 2.0 µL template, 2.0 µL dNTPs (2.5mM), 2.0 µL of 10X EX Taq buffer, 0.6 µL DMSO (3 %), 0.2 µL of EXTaq Hot Start polymerase (TaKaRa Bio Inc. Japan), and 11.20 µL ultra-pure water. The PCR reaction began with denaturation at 95 °C for 3 min; followed by 30 cycles at 94 °C for 20 s, 65 °C for 15 s, and 72 °C for 15 s; and a final extension at 72 °C for 5 min. The amplicon with the expected size (250–350 bp) was purified with the AccuPrep® Gel Purification Kit (Bioneer, Republic of Korea) after 1.5 % agarose gel electrophoresis. The purified amplicons were subjected to additional PCR to link each amplicon with the corresponding Nextera XT index. The second PCR mixture (20 µL) contained 5 µL template, 1 µL of a couple of index primers (10 pmol), 0.5 µL dNTPs (10 mM), 4 µL 5X Phusion HF Buffer, 8.3 µL ultrapure water, and 0.2 µL Phusion Hot Start Flex DNA polymerase (New England Biolabs, Hitchen, UK). The second PCR started at 94 °C for 5 min; followed by 15 cycles at 94 °C for 30 s, 55 °C for 30 s, and 72 °C for 30 s; and an additional 5 min at 72 °C. No noticeable bands were detected in the desired ranges for 16 field negative controls in 1.5 % agarose gel electrophoresis. Consequently, the 16 negative controls were discarded from the following analyses. After gel purification, the quality and quantity of the indexed PCR products with the expected sizes were analyzed using the Qubit dsDNAHS Assay Kit (Invitrogen, Carlsbad, CA, USA), followed by sequencing using the MiSeq platform (2 × 300 bp).

**Bioinformatic analysis of the NGS data**

The MiSeq raw reads were paired using Python 2.7 ([Zhang, 2015](#_ENREF_57)), and the paired reads were uploaded to the MiFish pipeline (<http://mitofish.aori.u-tokyo.ac.jp/mifish/>) for further analyses. In the MiFish pipeline, a low-quality tail of reads (QV ≤ 20) was trimmed in FASTQC. After taxonomic assignments from the MiFish pipeline, the sequences assigned to OTUs were compared with the GenBank database. If the sequence identity of the query sequence and top BLASTN hit was ≥ 99 %, the sequence was ascertained as a particular species. If the sequence identity ranged from 97 % to 99 %, the sequence was ascertained to the genus level, whereas sequences ranging from 97 % to 95 % were assigned as ‘unidentified’ genera. The geographic distribution of each species was assessed on the FishBase website (<https://www.fishbase.org/>). Alpha biodiversity was measured using the normalized read numbers from each sampling station of the four rivers sampled. The Shannon-Wiener (H') index indicates the heterogeneity of species or the richness of species in an ecosystem ([Gray, 2000](#_ENREF_16); [Magurran, 1988](#_ENREF_34)). The H′ index and the heat map clustering analysis were calculated using the PRIMER® v7 software ([Clarke and Gorley, 2015](#_ENREF_5)).

**RESULTS**

**Physicochemical parameters**

The water temperature of the sample sites ranged from 18.6 °C to 24.20 °C (Table 1). The Hyeongsan River showed the highest temperature difference (5.4 °C) between upstream (HS1) to downstream (HS4), whereas the lowest levels of temperature variation were observed in the Seomjin (0.8 ˚C) and Nakdong (1.5 ˚C) rivers. The lowest salinity (0.15 PSU) was measured at station 1 (upstream) of the Seomjin River, while the highest (20.20 PSU) was recorded at station 4 (downstream) of the Hyeongsan River. The salinity level increased from upstream to downstream in all rivers, except in the Nakdong River, where an artificial dam was constructed to block water from the ocean (Table 1).

**Analysis of fish haplotypes obtained using the MiFish pipeline**

The reliability of the MiFish pipeline (http://mitofish.aori.u-tokyo.ac.jp/mifish/workflows/new) for the biodiversity assessment of fish species inhabiting the sampled rivers was analyzed (Table 2). From 2,315,605 raw reads, 2,280,850 merged reads were obtained by the MiFish pipeline, with a 98.50 % yield from the raw reads. A total of 238 representative haplotypes were assigned to the default cutoff sequence identity. Among the 238 haplotypes, 125 unique haplotypes were identified using the phylogenetic tree analysis in the MEGA7 software ([Kumar et al., 2016](#_ENREF_31)) with a maximum likelihood algorithm (Fig. 2-5). A total of 2,241,130 reads (98.26 %) were assigned to 73 confirmed species, 46 genera, and 13 families of Teleostei, with 99 % as cutoff identity. The remaining 39,720 reads (49 haplotypes), which showed less than 99 % identity, were further assigned to 11 genera and eight unidentified genera (Table 3). A total of 34,755 reads (1.50 %) were discarded from further analyses. The highest species number was identified in the family Cyprinidae (35), followed by Gobiidae (11), and Cobitidae (8), while the remaining (19) were from other families of Teleostei. Among them, the highest species number (4 species) was identified in the genus *Acheilognathus*, followed by *Carassius, Misgurnus, Tridentiger*, and *Squalidus,* with three species in each of those genera (Table S1).

**Cyprinidae**

A total of 65 haplotypes were identified in the family Cyprinidae. Among the 65 haplotypes, 51 were assigned to 35 species of fishes with ≥ 99 % of sequence identity to the GenBank database (Fig. 2). Two haplotypes in the genus *Hemibarbus* from the Seomjin River (SJ1) and the Nakdong River (ND2) showed 100 % and 99 % identity to the sequences of *Hemibarbus labeo* (GenBank Number: DQ347953) and *Hemibarbus maculatus* (LC146032) sampled in Korea and Japan, respectively. Among the four endemic species in the genus *Hemibarbus*, *H. labeo* and *H. longirostris* are the most widely distributed species in Korea ([Lee et al., 2012](#_ENREF_33)). Two haplotypes identified from the Seomjin River (SJ1 and SJ2) and one from Taehwa River (TH1) showed 97 % and 95 % identity to a sequence of *H. longirostris* (LC049889), respectively, which suggests that these three haplotypes may be either *H. longirostris* or *H. mylodon* (Fig.2).

Five haplotypes were identified in the genus *Squalidus*. Four species of the genus have been reported from Korean waters: *Squalidus gracilis*, S. japonicus, S. multimaculatus, and S. chankaensis ([Kim and Park, 2002](#_ENREF_25)). Two haplotypes from the Taehwa (TH3) and Hyeongsan rivers (HS1) showed 100 % identity to sequences of S. japonicas coreanus (GenBank Number: KR075134) and S. multimaculatus (GenBank Number: KT948081), respectively. Another haplotype from the Hyeongsan River (HS3) showed 100% identity to a sequence of S. japonicas (GenBank Number: LC277782) sampled in Japan. Two haplotypes from the Seomjin River showed 99 % identity to a sequence of S. chankaensis tsuchigae (GenBank Number: KT948082) sampled in Korea.

Fishes of the subfamily Acheilognathinae, commonly known as bitterlings, deposit eggs in the gill cavities of freshwater mussels ([Kitamura, 2007](#_ENREF_27); [Kitamura et al., 2012](#_ENREF_28)). Approximately 60 species of bitterlings are considered valid in the genera *Acheilognathus*, *Tanakia*, and *Rhodeus* ([Arai, 1988](#_ENREF_1)). *Acheilognathus intermedia, A. macropterus, A. majusculus, A. rhombeus, Rhodeus suigensis, R. uyekii, Tanakia somjinensis,* and *T. signifier* were identified with a sequence identity > 99 % when compared to the GenBank database*.* Three haplotypes from the Seomjin River showed 99 % sequence identity to the respective haplotypes of *A. intermedia* (EF483933), *T. somjinensis* (FJ515921), and *T. signifier* (EF483930) sampled in Korea. Among them, *T. somjinensis* and *T. signifier* are endemic to Korea ([Kim and Park, 2002](#_ENREF_25)). One haplotype from the Taehwa River (TH3) showed 100 % identity to a sequence of *Rhynchocypris semotilus* (KT748874) sampled in Korea. This species is currently categorized as Critically Endangered in the Red Data Book of endangered fishes in Korea ([Ko et al., 2011](#_ENREF_30)).

Two sub-species of *Sarcocheilichthys* are known in Korea: *S. nigripinnis morii* and *S. variegatus wakiyae* ([Kim and Park, 2002](#_ENREF_25)). Two haplotypes from the Seomjin (SJ2) and Hyeongsan (HS2) rivers showed 100 % and 97 %, respectively, identity to a sequence of *S. variegatus wakiyae* (GenBank Number: KU301744) sampled in Korea. One haplotype from the Hyeongsan River (HS2) showed 100 % and 99.43 % identity to a sequence of *S. soldatovi* (LC146036) and the Korean haplotype of *S. nigripinnis morii* (AP017653) sampled in Japan and Korea, respectively. However, *S. soldatovi* is not currently reported for Korean waters. Therefore, further studies are needed to confirm the occurrence of this species in the Hyeongsan River for conservation purposes.

**Gobiidae**

We identified 16 haplotypes of the family Gobiidae, representing seven genera and 11 species (Fig. 3). Five haplotypes were identified in the genus *Tridentiger*, which represents the five known species of the genus recorded in Korea ([Kim et al., 2005](#_ENREF_26)). One haplotype from the Taehwa River (TH4) showed 100 % identity with a sequence of *T. obscures* (GenBank Number: KT601092) sampled in Korea. One haplotype from the Hyeongsan River (HS4) showed 100 % identity to a sequence of *T. trigonocephalus* (GenBank Number: LC385175) sampled in Japan, and another haplotype from the Seomjin River (SJ3) showed 100 % identity to a sequence of *T. trigonocephalus* (GenBank Number: KM030481) sampled in Korea. According to the recovered phylogenetic tree, the *T. trigonocephalus* haplotype from the Seomjin River is different from that of the Hyeongsan River (Fig. 3). All three haplotypes of the genus *Rhinogobius* showed 100 % identity to the database. The first and second haplotypes showed 100 % identity to sequences of *R. brunneus* sampled in Korea (KM030471) and Japan (LC049760), respectively. The third haplotype showed 100 % identity to a sequence of *R. giurinus* sampled in Korea (KM030475). Two haplotypes of *Gymnogobius* sp. from the Taehwa and Hyeongsan rivers showed 98 % sequence identity to *G. taranetzi* (GenBank Number: LC385155). Nine species of the genus *Gymnogobius* are currently reported in Korea ([Kim et al., 2005](#_ENREF_26)), and their MiFish sequences should be supplemented to the GenBank database.

**Cobitidae**

Sixteen species in five genera of the family Cobitidae are currently reported from Korean rivers ([Kim, 2009](#_ENREF_24)). A total of 18 haplotypes, representing five genera of the family, were identified (Fig. 4). Two haplotypes in the genus *Cobitis* identified in the Seomjin River were most closely related to *C. tetralineata* (LC146139) sampled in Japan, with 100 % and 99 % sequence identity. Two haplotypes from the Taehwa River showed 98 % and 97 % identity to *C. hankugensis* (LC146140). Two species of *Misgurnus* are reported from the Korean waters, *M. mizolepis* and *M. anguillicaudatus* ([Kim, 2009](#_ENREF_24)). Interestingly, two phylogenetically distinct clades in *M. anguillicaudatus* were identified in the phylogenetic analysis (Fig. 4). One of them was grouped with the haplotype of *M. bipartitus* (KF562047) sampled in China, while the other was clustered with the *M. mizolepis* (AP017654) sampled in Korea. *Misgurnus bipartitus* is currently reported to be endemic to China, and sequence data of Korean freshwater fishes in GenBank data should be reexamined.

Two haplotypes from the Hyeongsan River (HS1; KJ699181) and the Taehwa River (TH4; KM186182) showed 100 % identity with haplotypes of *Paramisgurnus dabryanus* sampled in China (Fig. 4). This species is regarded as endemic to China, but *P. dabryanus* is often imported to Korea together with *Misgurnus anguillicaudatus* due to their phenotypic similarity. Shimizu and Takagi (2010) concluded that there are different populations of *P. dabryanus* ([Shimizu and Takagi, 2010](#_ENREF_47)), and the two haplotypes of the species identified herein suggest that *P. dabryanus* has been imported from various locations in China. One haplotype from the Taehwa River (TH1) showed 100 % identity to a sequence of *Niwaella multifaciata* (EU670806) sampled in Korea, while another from the Hyeongsan River (HS1) showed a lower (96 %) identity to *Niwaella* sp. Therefore, further studies should be conducted to confirm the presence of species of this genus in the Hyeongsan River.

**Other families of Teleostei**

In addition to the three main families of Teleostei identified in this study, 27 additional haplotypes were found in the samples. These haplotypes represented 19 species belonging to14 genera and 11 families, namely Amblycipitidae (1), Anguillidae (1), Bagridae (5 haplotypes), Centrarchidae (3), Channidae (1), Clupeidae (2), Mugilidae (4), Odontobutidae (3), Pleuronectidae (1), Siluridae (3), and Sinipercidae (3). All the haplotypes of the family Bagridae were clearly identified and included: *Pseudobargrus ussuriensis*, *P. koreanus*, *Tachysurrus nitidus*, and *T. fulvidraco* (Fig. 5). Two species of *Silurus* are currently known in Korean rivers, *S. microdorsalis* and *S. asotus* ([Park and Kim, 1994](#_ENREF_38)). One haplotype from the Taehwa River (TH1) showed 99 % identity to a sequence of *Silurus microdorsalis* (GenBank Number: KT350610) sampled in Korea, whereas another haplotype from the Seomjin River (SJ1) showed a lower identity (96 %) with *S. microdorsalis* (KT350610) sampled in Korea.

One haplotype of the Amblycipitidae from the Seomjin River showed 97 % and 96 % identity to *Liobagrus styani* (KX096605) and *L. mediadiposalis* (KR075136), sampled in China and Korea, respectively. These results indicate that haplotypes of the family should be supplemented for accurate identification. Three species of *Odontobutis* are currently known in Korea: *O. interrupta*, *O. platycephala*, and *O. obscura* ([Kim et al., 2005](#_ENREF_26)). Two of them (*O. interrupta* and *O. platycephala*) were identified in this study with 100 % identity to the sequences of *O. interrupta* and *O. platycephala* sampled in Korea (KR364945 and KM030426). Two haplotypes of the genus *Coreoperca* showed 100 % and 97 % sequence identity to *Coreoperca herzi* (KR075132) sampled in Korea*.* Since two species of *Coreoperca* are reported to be endemic to the Korean peninsula ([Kim et al., 2005](#_ENREF_26)), the second haplotype is most likely *C. kawamebari*, but further studies should be conducted to confirm this identification. Two invasive species of the family Centrarchidae, the Bluegill (*Lepomis macrochirus*) and the Largemouth bass (*Micropterus salmoides*) were also identified in this study. These two species are endemic to North America but were introduced in the Korean peninsula for aquaculture purposes without considering their impact on local ecosystems.

**Fish biodiversity in the four rivers**

Fish assemblages in the four rivers included in this study were analyzed. Among the 73 confirmed fish species detected in this study, 13 were identified in all four rivers: *Anguilla japonica, Hemibarbus labeo, Konosirus punctatus, Micropterus salmoides, Misgurnus mizolepis, Mugil cephalus, Opsariichthys uncirostris, Pseudorasbora parva, Rhinogobiu sbrunneus, Rhynchocypris lagowskii, Silurus asotus, Tridentiger obscurus,* and *Zacco platypus* (Fig. 6). Regardless of sample stations, species of the Cyprinidae appear to be dominant, with average proportions of 47.02 ± 6.73 %, followed by the Gobiidae (15.24 ± 3.07 %) and Cobitidae (9.95 ± 4.09 %; Fig.7). However, the proportions of species in those families were different between upstream and downstream stations. The proportion of Cyprinidae species was higher (45.27 ± 9.1 %) upstream (stations 1 and 2) than downstream (33.78 ± 18 % at station 4). In contrast, the proportion of Gobiidae was lower (14.53 ± 8.28 %) upstream than downstream (station 4, 19.90 ± 14 %).

The highest number of species was recorded in the Seomjin River (52 species), followed by the Taehwa (42 species), Hyeongsan (40 species), and Nakdong (26 species) rivers. A total of 17 species were exclusively recorded in the Seomjin River: *Acanthogobius hasta, Acheilognathus intermedia, A. majusculus, A. rhombeus, Cobitis tetralineata, Coreoleuciscus splendidus, Kareius bicoloratus, Microphysogobio yaluensis, Phoxinus oxycephalus, Pseudobagrus koreanus, Rhodeus suigensis, R. uyekii, Sarcocheilichthys variegatus, Siniperca scherzeri, Squalidus gracilis, Tanakia somjinensis,* and *T. signifier.* Five species were only recorded in the Taehwa River: *Acanthogobius lactipes, Mugilogobius abei, Pseudogobius masago, Rhynchocypris semotilus,* and *Silurus microdorsalis*, whereas four species were only identified in the Nakdong River: *Plagiognathops microlepis*, *Pseudobagrus ussuriensis, Rhinogobius giurinus,* and *Tachysurus nitidus.* Finally, only three species (*Nipponocypris koreanus, Sarcocheilichthys soldatovi,* and *Squalidus multimaculatus*) were exclusively recorded in the Hyeongsan River (Fig. 6).

The highest Shannon index (SI) was identified in the Seomjin River (3.480), followed by the Taehwa (3.067), Hyeongsan (2.954), and Nakdong (2.864) rivers. Among the 16 surveyed stations, station 1 of the Seomjin River (SJ1) showed the highest species richness (2.197), whereas the lowest richness (1.008) was recorded at the station 4 of the Nakdong River (ND4). From upstream to downstream, average species richness decreased from 1.951 to 1.415 (Table 4).

**Clustering analysis**

In order to assess the correlation between the fish assemblage and sample stations, we conducted a heat-map analysis with the 30 most abundant species using Primer software ([Clarke and Gorley, 2015](#_ENREF_5)). The results indicate the species distribution in different sampling stations (Fig. 8). In upstream sites (Stations 1 and 2), the dominant species were *A. intermedia, Coreoperca herzi, Misgurnus mizolepis, Nipponocypris temminckii, Rhynchocypris lagowskii, Odontobutis interrupta, O. platycephala, Tanakia signifier,* and *Zacco platypus*. At station 3, the dominant species were *Gymnogobius breunigii, Mugil cephalus, Pseudorasbora parva, Rhinogobius giurinus,* and *R. brunneus*. Finally, in the downstream sample (Station 4), *Anguilla japonica, Konosirus punctatus, Mugil cephalus, Planiliza haematocheila, Tridentiger obscurus,* and *T. trigonocephalus* were identified as the dominant species, all of which were either euryhaline or anadromous (<https://www.fishbase.org>).

**DISCUSSION**

The results indicate that eDNA metabarcoding using the MiFish pipeline is a useful tool for assessing fish biodiversity in Korean freshwater ecosystems, since a total of 125 unique haplotypes, including at least 73 species, were successfully identified by a single-day survey of 16 sampling stations in four rivers (Fig. 2–5). According to the “Survey and Evaluation of Aquatic Ecosystem Health (SEAEH),” a total of 130 freshwater fish species were identified from 953 sampling sites that covered most of the Korean rivers and lakes ([Yoon et al., 2012](#_ENREF_56)). The total number of species confirmed by eDNA metabarcoding was equivalent to approximately 56 % of those obtained by the year-long conventional surveys. The efficiency of eDNA metabarcoding might actually be even higher, especially considering the number of haplotypes successfully identified at the genus and/or family level. This result indicates that eDNA metabarcoding with the MiFish pipeline can significantly contribute to the assessment of freshwater fish biodiversity in Korea, especially considering its relatively lower cost of implementation when compared with more conventional morphology-based surveys. Although the methodology in each research group may be slightly different, similar conclusions have been reached in other studies ([Bista et al., 2017](#_ENREF_3); [Deiner et al., 2016](#_ENREF_10)). eDNA metabarcoding analysis is also adequate for surveying aquatic species in protected areas, as it minimizes disturbance of vulnerable communities ([Fernandez et al., 2018](#_ENREF_13)).

Despite its relevance as a methodology for the assessment of biodiversity, there are still a few shortcomings for a more widespread use of eDNA metabarcoding by the MiFish pipeline. First, MiFish sequence data for endemic species of Korea should be supplemented to the GenBank database. According to the Archive of Korean species (<https://species.nibr.go.kr>), 67 species of freshwater fishes are endemic to Korea, and many of their MiFish sequences are still not available in the GenBank database. In addition to the lack of sequence data, freshwater fishes typically have intra-species genetic distances that are generally higher than those of marine species ([Seehausen and Wagner, 2014](#_ENREF_44)). Second, the MiFish primer amplifies the 12S rRNA gene (163–185 bp) region of mitochondrial DNA, which is smaller and less variable than the COI region, which is typically used in species identification ([IVANOVA et al., 2007](#_ENREF_18)). In fact, the MiFish region was unable to differentiate several closely related marine fish taxa, such as those in the genus *Sebastes* and *Takifugu* ([Sato et al., 2018](#_ENREF_43); [Yamamoto et al., 2017](#_ENREF_53)). We also found that the average genetic distance of several genera in the family Cyprinidae was low in the MiFish region. For example, the average genetic distance of *Carassius* species was too low (0.01) and the identification at the species level was not possible (Fig. 2).

Further studies using eDNA metabarcoding might also be relevant to obtain more than biodiversity data, such as the quantitative analysis of fish species. It is difficult to estimate the spatial abundance of eDNA in lotic environments. In fact, many factors should be considered for the quantitative analysis of eDNAs in rivers, including water dynamics ([Deiner and Altermatt, 2014](#_ENREF_9); [Jerde et al., 2016](#_ENREF_21); [Wilcox et al., 2016](#_ENREF_51)) or different decaying times due to different physical, chemical, or biological factors ([Shapiro, 2008](#_ENREF_45)). It is generally known that shorter fragments of DNA are degraded slower than larger ones, increasing their probability of detection in natural environments ([Deagle et al., 2006](#_ENREF_8)). Therefore, it is still too early to adopt eDNA metabarcoding for the quantitative analysis of fish species under natural conditions. For the quantitative study, standardized collection methods and pretreatment procedures for NGS sequencing analysis should also be established. One of the strongest points in the biodiversity survey by eDNA metabarcoding is the quantity of information it can generate compared with more conventional surveys since large datasets are useful for statistical analyses. However, large amounts of data have been produced using different water collection methods, eDNA preparation, sequencing, and bioinformatic analysis platforms by different research groups in different countries. Therefore, the interconversion of data is currently not possible. The establishment of an international standard regarding the overall methodology of eDNA metabarcoding would help researchers to produce more comparable data.

According to the results obtained in this study, the highest species richness was found in the Seomjin River (3.48) compared with those of the other three rivers: the Taehwa River (3.06), Hyeongsan River (2.95), and Nakdong River (2.86). The lower values of species richness detected in the Nakdong, Hyeongsan, and Taehwa rivers are presumably related to the higher anthropogenic alteration of the natural conditions in those rivers. Like most other Korean rivers, these three rivers run through highly populated metropolitan cities, in which rivers are exposed to various human impacts that directly or indirectly promote changes in the diversity and distribution of freshwater fishes ([Finkenbine et al., 2000](#_ENREF_14)). In particular, the lowest species richness (2.86) and number of endemic species (only one, *Odontobutis interrupta*) were identified in the Nakdong River, where the highest number of constructions and population exist among the sampled rivers. Lee et al. (2015) reported only two endemic species (*Coreoperca herzi* and *Odontobutis platycephala*) in the Nakdong River using a conventional catch survey. Moreover, eight endemic species (*Coreoleuciscus splendidus, Iksookimia longicorpa, Microphysogobio koreensis, M. yaluensis, Odontobutis interrupta, O. platycephala, Pseudobagrus koreanus,* and *Squalidus gracilis*) were identified in this study in the Seomjin River, a number that is similar to those obtained in previous studies ([Jang et al., 2003](#_ENREF_19); [Lee et al., 2015](#_ENREF_32)). Several constructions along urbanized watersheds, including dams and weirs, have caused the simplification and reduction of habitats, decreasing the biodiversity in the river ([Nilsson et al., 2005](#_ENREF_37); [Riley et al., 2005](#_ENREF_42)). In contrast, there is no metropolitan city along the Seomjin River, which is, therefore, less exposed to anthropogenic impacts. A long-term survey should be conducted to establish a clear correlation between anthropogenic factors and fish assemblages in the Korean rivers.

The eDNA metabarcoding analysis also indicates that some exotic fish species are widely distributed in Korean rivers. We were able to identify at least five exotic fish species: *Carassius cuvieri, Cyprinus carpio, C. megalophthalmus, Lepomis macrochirus*, and *Micropterus salmoides* (Table S3). These exotic species may affect native fishes in terms of shelter and spawning sites. They can also disturb the food chain, preying on native fish. In addition, these species have a high reproductive capacity, which makes them important potentially invasive species (Keller & Lake, 2007; Koster *et al.*, 2002; Nico & Fuller, 2010). Surprisingly, our results also revealed that the largemouth bass, *M. salmoides*, and the bluegill, *L. macrochirus*, are likely present in all the sampled rivers. These two species, which are native to North America, were artificially introduced in the 1970s in Korea as freshwater fish stock, without any further consideration of the effects on the freshwater ecosystems of the country. They are now widely distributed throughout the Korean Peninsula, competing with the native species. A long-term survey of these rivers should be conducted to properly assess the potential impacts of these introduced species ([Jang et al., 2002](#_ENREF_20); [Yoon et al., 2012](#_ENREF_56)). Freshwater ecosystems are much more vulnerable to invasive species, causing biodiversity loss and global climate change ([Clavero and García-Berthou, 2005](#_ENREF_6)), and eDNA metabarcoding analyses would be useful for monitoring the distribution patterns of invasive species in Korean rivers.

**Acknowledgments**

The authors are thankful to the Ministry of Oceans and Fisheries of the Republic of Korea. The authors also thank the reviewers for their valuable comments and suggestions for the manuscript.

**Additional information and declarations**

**Funding:**

This research was a part of the project titled “Long-term change of structure and function in marine ecosystems of Korea”, funded by the Ministry of Oceans and Fisheries, Korea.

**Role of funding**

The funding sources had no role in the research design, sample collection, data analysis, manuscript writing, or the decision to submit the article for publication.

**Competing Interests**

The authors declare that they have no competing interests.

**Author Contributions**

- Md. JobaidulAlam collected the samples, performed the experiments, analyzed the data, prepared figures and/or tables, and wrote the manuscript.
- Nack-KeunKim collected the samples and analyzed the data
- SaptoAndriyono performed the experiments, analyzed the data, prepared figures, and/or tables
- Hee-KyuChoi analyzed the data, prepared figures, and/or tables
- Ji-Hyun Lee analyzed the data, prepared figures, and/or tables
- Hyun-Woo Kim conceived and designed the experiments, analyzed the data, contributed reagents/materials/analysis tools, authored or reviewed drafts of the manuscript, approved the final draft, and wrote the manuscript.

**REFERENCES**

Arai, R., 1988. Acheilognathus melanogaster, a senior synonym of A moriokae, with a revision of the genera of the subfamily Acheilogathinae (Cypriniformes, Cyprinidae). Bul Nat Sci Mus Tokyo Ser A 14, 199-213.

Ardura, A., Zaiko, A., Martinez, J.L., Samulioviene, A., Semenova, A., Garcia-Vazquez, E., 2015. eDNA and specific primers for early detection of invasive species–a case study on the bivalve Rangia cuneata, currently spreading in Europe. Marine Environmental Research 112, 48-55.

Bista, I., Carvalho, G.R., Walsh, K., Seymour, M., Hajibabaei, M., Lallias, D., Christmas, M., Creer, S., 2017. Annual time-series analysis of aqueous eDNA reveals ecologically relevant dynamics of lake ecosystem biodiversity. Nature communications 8, 14087.

Cai, W., Ma, Z., Yang, C., Wang, L., Wang, W., Zhao, G., Geng, Y., Douglas, W.Y., 2017. Using eDNA to detect the distribution and density of invasive crayfish in the Honghe-Hani rice terrace World Heritage site. PloS one 12, e0177724.

Clarke, K., Gorley, R., 2015. Getting started with PRIMER v7. PRIMER-E: Plymouth, Plymouth Marine Laboratory.

Clavero, M., García-Berthou, E., 2005. Invasive species are a leading cause of animal extinctions. Trends in Ecology & Evolution 20, 110.

Clusa, L., Miralles, L., Basanta, A., Escot, C., García-Vázquez, E., 2017. eDNA for detection of five highly invasive molluscs. A case study in urban rivers from the Iberian Peninsula. PloS one 12, e0188126.

Deagle, B.E., Eveson, J.P., Jarman, S.N., 2006. Quantification of damage in DNA recovered from highly degraded samples–a case study on DNA in faeces. Frontiers in zoology 3, 11.

Deiner, K., Altermatt, F., 2014. Transport distance of invertebrate environmental DNA in a natural river. PloS one 9, e88786.

Deiner, K., Fronhofer, E.A., Mächler, E., Walser, J.-C., Altermatt, F., 2016. Environmental DNA reveals that rivers are conveyer belts of biodiversity information. Nature communications 7, 12544.

Dejean, T., Valentini, A., Miquel, C., Taberlet, P., Bellemain, E., Miaud, C., 2012. Improved detection of an alien invasive species through environmental DNA barcoding: the example of the American bullfrog Lithobates catesbeianus. Journal of applied ecology 49, 953-959.

Dudgeon, D., 2010. Prospects for sustaining freshwater biodiversity in the 21st century: linking ecosystem structure and function. Current Opinion in Environmental Sustainability 2, 422-430.

Fernandez, S., Sandin, M.M., Beaulieu, P.G., Clusa, L., Martinez, J.L., Ardura, A., García-Vázquez, E., 2018. Environmental DNA for freshwater fish monitoring: insights for conservation within a protected area. PeerJ 6, e4486.

Finkenbine, J.K., Atwater, J., Mavinic, D., 2000. STREAM HEALTH AFTER URBANIZATION 1. JAWRA Journal of the American Water Resources Association 36, 1149-1160.

Foote, A.D., Thomsen, P.F., Sveegaard, S., Wahlberg, M., Kielgast, J., Kyhn, L.A., Salling, A.B., Galatius, A., Orlando, L., Gilbert, M.T.P., 2012. Investigating the potential use of environmental DNA (eDNA) for genetic monitoring of marine mammals. PloS one 7, e41781.

Gray, J.S., 2000. The measurement of marine species diversity, with an application to the benthic fauna of the Norwegian continental shelf. Journal of experimental marine biology and ecology 250, 23-49.

Gustavson, M., Collins, P., Finarelli, J., Egan, D., Conchúir, R., Wightman, G., King, J., Gauthier, D., Whelan, K., Carlsson, J., 2015. An eDNA assay for Irish Petromyzon marinus and Salmo trutta and field validation in running water. Journal of Fish Biology 87, 1254-1262.

IVANOVA, N.V., ZEMLAK, T.S., HANNER, R.H., HEBERT, P.D.N., 2007. Universal primer cocktails for fish DNA barcoding. Molecular Ecology Notes 7, 544-548.

Jang, M.-H., Lucas, M.C., Joo, G.-J., 2003. The fish fauna of mountain streams in South Korean national parks and its significance to conservation of regional freshwater fish biodiversity. Biological Conservation 114, 115-126.

Jang, M.H., Kim, J.G., Park, S.B., Jeong, K.S., Cho, G.I., Joo, G.J., 2002. The current status of the distribution of introduced fish in large river systems of South Korea. International Review of Hydrobiology 87, 319-328.

Jerde, C.L., Olds, B.P., Shogren, A.J., Andruszkiewicz, E.A., Mahon, A.R., Bolster, D., Tank, J.L., 2016. Influence of stream bottom substrate on retention and transport of vertebrate environmental DNA. Environmental science & technology 50, 8770-8779.

Kelly, R.P., Closek, C.J., O'Donnell, J.L., Kralj, J.E., Shelton, A.O., Samhouri, J.F., 2017. Genetic and manual survey methods yield different and complementary views of an ecosystem. Frontiers in Marine Science 3, 283.

Kelly, R.P., Port, J.A., Yamahara, K.M., Martone, R.G., Lowell, N., Thomsen, P.F., Mach, M.E., Bennett, M., Prahler, E., Caldwell, M.R., 2014. Harnessing DNA to improve environmental management. Science 344, 1455-1456.

Kim, I.-S., 2009. A Review of the Spined Loaches, Family Cobitidae (Cypriniformes) in Korea Korean Journal of Ichthyology 21, 7-28.

Kim, I.-S., Park, J.-Y., 2002. Freshwater fishes of Korea. Kyo hak sa.

Kim, I., Choi, Y., Lee, C., Lee, Y., Kim, B., Kim, J., 2005. Illustrated book of Korean fishes. Kyo-Hak Publishing, 417-418.

Kitamura, J.-i., 2007. Reproductive ecology and host utilization of four sympatric bitterling (Acheilognathinae, Cyprinidae) in a lowland reach of the Harai River in Mie, Japan. Environmental Biology of Fishes 78, 37-55.

Kitamura, J., Nagata, N., Nakajima, J., Sota, T., 2012. Divergence of ovipositor length and egg shape in a brood parasitic bitterling fish through the use of different mussel hosts. Journal of evolutionary biology 25, 566-573.

Klymus, K.E., Marshall, N.T., Stepien, C.A., 2017. Environmental DNA (eDNA) metabarcoding assays to detect invasive invertebrate species in the Great Lakes. PloS one 12, e0177643.

Ko, M., Kim, K., Park, J., 2011. Red Data Book of endangered fishes in Korea. National Institute of Biological Resources, Incheon.(in Korean).

Kumar, S., Stecher, G., Tamura, K., 2016. MEGA7: molecular evolutionary genetics analysis version 7.0 for bigger datasets. Molecular biology and evolution 33, 1870-1874.

Lee, J.W., Yoon, J.D., Kim, J.H., Park, S.H., Baek, S.H., Yu, J.J., Jang, M.H., Min, J.I., 2015. Length‐weight relationships for 18 freshwater fish species from the Nakdong River in South Korea. Journal of Applied Ichthyology 31, 576-577.

Lee, W.-O., Zhang, M.-M., Oh, C.-W., Baek, J.-M., Song, K.-J., 2012. Age and Growth of Barbel Steed Hemibarbus labeo in Goe-san Lake in Korea. Fisheries and aquatic sciences 15, 353-359.

Magurran, A.E., 1988. Ecological diversity and its measurement. Princeton university press.

Miya, M., Sato, Y., Fukunaga, T., Sado, T., Poulsen, J.Y., Sato, K., Minamoto, T., Yamamoto, S., Yamanaka, H., Araki, H., 2015a. MiFish, a set of universal PCR primers for metabarcoding environmental DNA from fishes: detection of more than 230 subtropical marine species. Royal Society open science 2, 150088.

Miya, M., Sato, Y., Fukunaga, T., Sado, T., Poulsen, J.Y., Sato, K., Minamoto, T., Yamamoto, S., Yamanaka, H., Araki, H., Kondoh, M., Iwasaki, W., 2015b. MiFish, a set of universal PCR primers for metabarcoding environmental DNA from fishes: detection of more than 230 subtropical marine species. Royal Society Open Science 2.

Nilsson, C., Reidy, C.A., Dynesius, M., Revenga, C., 2005. Fragmentation and flow regulation of the world's large river systems. Science 308, 405-408.

Park, S.-W., Kim, Y.-G., 1994. Studies on disease of catfish, Silurus asotus, in Korea. III. Edwardsiella ictaluri infection. Journal of fish pathology 7, 105-112.

Pilliod, D.S., Goldberg, C.S., Laramie, M.B., Waits, L.P., 2013. Application of environmental DNA for inventory and monitoring of aquatic species. US Department of the Interior, US Geological Survey.

Pont, D., Rocle, M., Valentini, A., Civade, R., Jean, P., Maire, A., Roset, N., Schabuss, M., Zornig, H., Dejean, T., 2018. Environmental DNA reveals quantitative patterns of fish biodiversity in large rivers despite its downstream transportation. Scientific reports 8, 10361.

Riaz, T., Shehzad, W., Viari, A., Pompanon, F., Taberlet, P., Coissac, E., 2011. ecoPrimers: inference of new DNA barcode markers from whole genome sequence analysis. Nucleic Acids Research 39, e145-e145.

Riley, S.P., Busteed, G.T., Kats, L.B., Vandergon, T.L., Lee, L.F., Dagit, R.G., Kerby, J.L., Fisher, R.N., Sauvajot, R.M., 2005. Effects of urbanization on the distribution and abundance of amphibians and invasive species in southern California streams. Conservation Biology 19, 1894-1907.

Sato, Y., Miya, M., Fukunaga, T., Sado, T., Iwasaki, W., 2018. MitoFish and MiFish Pipeline: A Mitochondrial Genome Database of Fish with an Analysis Pipeline for Environmental DNA Metabarcoding. Molecular Biology and Evolution 35, 1553-1555.

Seehausen, O., Wagner, C.E., 2014. Speciation in Freshwater Fishes. Annual Review of Ecology, Evolution, and Systematics 45, 621-651.

Shapiro, B., 2008. Engineered polymerases amplify the potential of ancient DNA. Trends in biotechnology 26, 285-287.

Shaw, J.L.A., Clarke, L.J., Wedderburn, S.D., Barnes, T.C., Weyrich, L.S., Cooper, A., 2016. Comparison of environmental DNA metabarcoding and conventional fish survey methods in a river system. Biological Conservation 197, 131-138.

Shimizu, T., Takagi, M., 2010. Two genetic clades in populations of Paramisgurnus dabryanus, an exotic invader in ehime prefecture. Jpn J Ichthyol 57, 125-134.

Stoeckle, M.Y., Soboleva, L., Charlop-Powers, Z., 2017. Aquatic environmental DNA detects seasonal fish abundance and habitat preference in an urban estuary. PLOS ONE 12, e0175186.

Takahara, T., Minamoto, T., Doi, H., 2013. Using environmental DNA to estimate the distribution of an invasive fish species in ponds. PloS one 8, e56584.

Ushio, M., Murakami, H., Masuda, R., Sado, T., Miya, M., Sakurai, S., Yamanaka, H., Minamoto, T., Kondoh, M., 2017. Quantitative monitoring of multispecies fish environmental DNA using high-throughput sequencing. bioRxiv.

Wilcox, T.M., McKelvey, K.S., Young, M.K., Sepulveda, A.J., Shepard, B.B., Jane, S.F., Whiteley, A.R., Lowe, W.H., Schwartz, M.K., 2016. Understanding environmental DNA detection probabilities: A case study using a stream-dwelling char Salvelinus fontinalis. Biological Conservation 194, 209-216.

Williams, K.E., Huyvaert, K.P., Vercauteren, K.C., Davis, A.J., Piaggio, A.J., 2018. Detection and persistence of environmental DNA from an invasive, terrestrial mammal. Ecology and evolution 8, 688-695.

Yamamoto, S., Masuda, R., Sato, Y., Sado, T., Araki, H., Kondoh, M., Minamoto, T., Miya, M., 2017. Environmental DNA metabarcoding reveals local fish communities in a species-rich coastal sea. Scientific reports 7, 40368.

Yamamoto, S., Minami, K., Fukaya, K., Takahashi, K., Sawada, H., Murakami, H., Tsuji, S., Hashizume, H., Kubonaga, S., Horiuchi, T., 2016. Environmental DNA as a ‘snapshot’of fish distribution: A case study of Japanese jack mackerel in Maizuru Bay, Sea of Japan. PLoS One 11, e0149786.

Yamanaka, H., Minamoto, T., 2016. The use of environmental DNA of fishes as an efficient method of determining habitat connectivity. Ecological indicators 62, 147-153.

Yoon, J.-D., Jang, M.-H., Kim, H.-W., Joo, G.-J., 2012. Fish Biodiversity Monitoring in Rivers of South Korea, The Biodiversity Observation Network in the Asia-Pacific Region. Springer, 175-191.

Zhang, Y., 2015. An Introduction to Python and computer programming, An Introduction to Python and Computer Programming. Springer, 1-11.
