# Supplementary material for: Assessment of fish biodiversity in four Korean rivers using environmental DNA metabarcoding
Source: PeerJ. 2020 Jul 14;8:e9508. doi: 10.7717/peerj.9508 (PMC7367050; doi:10.7717/peerj.9508)
Supplement: Table S1 [file peerj-08-9508-s002.docx]

**Table S1: List of fish species with the GenBank numbers identified from the eDNA metabarcoding study of the four rivers**

| No. | Family | Haplotypes | Identity (%) | Korean haplotype | Chinese haplotype | Japanese haplotype | Others |
| --- | --- | --- | --- | --- | --- | --- | --- |
| 1 | Gobiidae | *Acanthogobius hasta* | 100 | KM030428 | KM891736 | - |  |
| 2 | Gobiidae | *Acanthogobius lactipes* | 100 | KM030431 | - | LC385140 |  |
| 3 | Cyprinidae | *Acheilognathus intermedia* | 99 | EF483933 | - | - |  |
| 4 | Cyprinidae | *Acheilognathus macropterus* | 99 | EF483935 | KJ499466 | LC092100 |  |
| 5 | Cyprinidae | *Acheilognathus majusculus* | 99 | - | - | LC006056 |  |
| 6 | Cyprinidae | *Acheilognathus rhombeus* | 99 | KT601094 | - | LC146100 |  |
| 7 | Cyprinidae | *Acheilognathus* sp. (unidentified) | 95 |  |  | LC006056 |  |
| 8 | Anguillidae | *Anguilla japonica* | 100 | HQ185628 | MH050933 | LC193417 |  |
| 9 | Cyprinidae | *Carassius auratus* | 100 | - | KX505165 |  |  |
| 10 | Cyprinidae | *Carassius auratus* | 100 |  |  |  | Turkey KM657132 |
| 11 | Cyprinidae | *Carassius auratus* | 99 |  | AY771781 | LC193299 |  |
| 12 | Cyprinidae | *Carassius auratus* | 99 | - | AY771781 | LC193299 |  |
| 13 | Cyprinidae | *Carassius cuvieri* | 100 | - | - | AP011237 |  |
| 14 | Cyprinidae | *Carassius cuvieri* | 100 |  |  | AP011237 |  |
| 15 | Channidae | *Channa argus* | 100 | - | MG751766 | AB972107 |  |
| 16 | Cobitidae | *Cobitis* sp. | 97 | EU670794 | - | LC146139 |  |
| 17 | Cobitidae | *Cobitis* sp. | 97 | EU670794 | - | LC146139 |  |
| 18 | Cobitidae | *Cobitis tetralineata* | 100 | EU670794 | - | LC146139 |  |
| 19 | Cobitidae | *Cobitis tetralineata* | 99 | EU670794 | - | LC146139 |  |
| 20 | Cyprinidae | *Coreoleuciscus* sp. (unidentified) | 96 | JN831358 | - | AP011258 |  |
| 21 | Cyprinidae | *Coreoleuciscus splendidus* | 100 | JN831358 | - | AP011258 |  |
| 22 | Sinipercidae | *Coreoperca herzi* | 100 | KR075132 | - | - |  |
| 23 | Sinipercidae | *Coreoperca* sp. | 97 | KR075132 | - | - |  |
| 24 | Cyprinidae | *Cyprinus carpio* | 100 | - | KX710076 | AP017363 |  |
| 25 | Cyprinidae | *Cyprinus carpio* | 100 | - | KX710076 | AP017363 |  |
| 26 | Cyprinidae | *Cyprinus carpio* | 99 | - | KX710076 | AP017363 |  |
| 27 | Cyprinidae | *Cyprinus megalophthalmus* | 100 | - | KR869143 | - |  |
| 28 | Gobiidae | *Favonigobius gymnauchen* | 100 | - | - | LC385206 |  |
| 29 | Gobiidae | *Gymnogobius breunigii* | 99 | KM030451 | - | - |  |
| 30 | Gobiidae | *Gymnogobius* sp. | 98 | KM030451 | - | - |  |
| 31 | Gobiidae | *Gymnogobius* sp. | 98 | KM030451 | - | - |  |
| 32 | Cyprinidae | *Hemibarbus labeo* | 100 | DQ347953 | KP064328 | LC049898 |  |
| 33 | Cyprinidae | *Hemibarbus maculatus* | 99 | - | NC018534 |  |  |
| 34 | Cyprinidae | *Hemibarbus* sp. | 97 | DQ347953 | KP064328 | LC049898 |  |
| 35 | Cyprinidae | *Hemibarbus* sp. | 97 | DQ347953 | KP064328 | LC049898 |  |
| 36 | Cyprinidae | *Hemibarbus* sp. (unidentified) | 95 | DQ347953 | KP064328 | LC049898 |  |
| 37 | Cyprinidae | *Hemiculter leucisculus* | 100 | - | - | LC340359 |  |
| 38 | Cobitidae | *Iksookimia longicorpa* | 100 | KM676413 | - | LC146135 |  |
| 39 | Cobitidae | *Iksookimia yongdokensis* | 100 | EU670800 | - | - |  |
| 40 | Cobitidae | *Iksookimia yongdokensis* | 99 | EU670800 | - | - |  |
| 41 | Pleuronectidae | *Kareius bicoloratus* | 100 | - | - | AP002951 |  |
| 42 | Clupeidae | *Konosirus punctatus* | 100 | - | KC477844 | LC020951 | Taiwan AP011612 |
| 43 | Clupeidae | *Konosirus punctatus* | 99 | - | KC477844 | LC020951 | Taiwan AP011612 |
| 44 | Centrarchidae | *Lepomis macrochirus* | 100 | - | JN389795 | AP005993 | USA KP013118 |
| 45 | Amblycipitidae | *Liobagrus* sp. | 97 | KR075136 | KX096605 | AP012015 |  |
| 46 | Cyprinidae | *Microphysogobio koreensis* | 100 | FJ515920 | - | - |  |
| 47 | Cyprinidae | *Microphysogobio yaluensis* | 99 | KR075133 | - | AP012073 |  |
| 48 | Centrarchidae | *Micropterus salmoides* | 100 | - | HQ391896 | LC069536 | USA DQ536425 |
| 49 | Centrarchidae | *Micropterus salmoides* | 99 | - | HQ391896 | LC069536 | USA DQ536425 |
| 50 | Cobitidae | *Misgurnus anguillicaudatus* | 100 | - | KC762740 | - |  |
| 51 | Cobitidae | *Misgurnus anguillicaudatus* | 99 | - | KC762740 | - |  |
| 52 | Cobitidae | *Misgurnus anguillicaudatus* | 99 | EU670804 | - | - |  |
| 53 | Cobitidae | *Misgurnus anguillicaudatus* | 99 | - | - | LC385093 |  |
| 54 | Cobitidae | *Misgurnus bipartitus* | 100 | - | KF562047 | LC091592 |  |
| 55 | Cobitidae | *Misgurnus mizolepis* | 100 | AP017654 | - | - |  |
| 56 | Cobitidae | *Misgurnus mizolepis* | 99 | AP017654 | - | - |  |
| 57 | Mugilidae | *Mugil cephalus* | 100 | - | KF374974 | LC278014 |  |
| 58 | Gobiidae | *Mugilogobius abei* | 100 | KM030465 | - | LC421743 | Taiwan KF128984 |
| 59 | Cyprinidae | *Nipponocypris koreanus* | 100 | - | KJ427719 | - |  |
| 60 | Cyprinidae | *Nipponocypris temminckii* | 100 | - | - | AP012116 |  |
| 61 | Cobitidae | *Niwaella multifasciata* | 100 | EU670807 | - | LC146133 |  |
| 62 | Cobitidae | *Niwaella* sp. (unidentified) | 96 | EU670807 | - | LC146133 |  |
| 63 | Odontobutidae | *Odontobutis interrupta* | 100 | KR364945 | - | - |  |
| 64 | Odontobutidae | *Odontobutis platycephala* | 100 | KM030426 | - | - |  |
| 65 | Odontobutidae | *Odontobutis platycephala* | 99 | KM030426 |  |  |  |
| 66 | Cyprinidae | *Opsariichthys* sp. (unidentified) | 96 | - | - | AB218897 |  |
| 67 | Cyprinidae | *Opsariichthys uncirostris* | 99 | - | - | AB218897 |  |
| 68 | Cobitidae | *Paramisgurnus dabryanus* | 100 | - | KM186182 | LC146125 |  |
| 69 | Cobitidae | *Paramisgurnus dabryanus* | 100 | - | KJ699181 | LC146125 |  |
| 70 | Cyprinidae | *Phoxinus oxycephalus* | 99 | MK208924 | - | AB626852 |  |
| 71 | Cyprinidae | *Phoxinus oxycephalus* | 99 | MK208924 | - | AB626852 |  |
| 72 | Cyprinidae | *Phoxinus semotilus* | 100 | KT748874 | - | - |  |
| 73 | Mugilidae | *Planiliza affinis* | 100 | - | KM925142 | LC277843 |  |
| 74 | Mugilidae | *Planiliza haematocheila* | 100 | - | KJ622047 | LC021099 |  |
| 75 | Mugilidae | *Planiliza haematocheila* | 100 | - | KJ622047 | LC021099 |  |
| 76 | Bagridae | *Pseudobagrus koreanus* | 100 | KT601095 | - | - |  |
| 77 | Bagridae | *Pseudobagrus ussuriensis* | 100 | - | KC188782 | - |  |
| 78 | Bagridae | *Pseudobagrus ussuriensis* | 99 | - | KC188782 | - |  |
| 79 | Cyprinidae | *Pseudogobio esocinus* | 100 | - | - | LC340042 |  |
| 80 | Cyprinidae | *Pseudogobio esocinus* | 99 | - | - | LC340042 |  |
| 81 | Cyprinidae | *Pseudogobio vaillanti* | 100 | - | KU314695 | LC146041 |  |
| 82 | Cyprinidae | *Pseudogobio vaillanti* | 99 | - | KU314695 | LC146041 |  |
| 83 | Gobiidae | *Pseudogobius masago* | 100 | KM030467 | - | LC049791 |  |
| 84 | Cyprinidae | *Pungtungia herzi* | 99 | KF006339 | - | AB239598 |  |
| 85 | Cyprinidae | *Pungtungia* sp. | 97 | KF006339 | - | AB239598 |  |
| 86 | Cyprinidae | *Pungtungia* sp. (unidentified) | 96 | KF006339 | - | AB239598 |  |
| 87 | Gobiidae | *Rhinogobius brunneus* | 100 | KT601096 | - |  |  |
| 88 | Gobiidae | *Rhinogobius brunneus* | 100 |  |  | LC049760 |  |
| 89 | Gobiidae | *Rhinogobius giurinus* | 100 | KM030475 | KP892753 | LC049748 |  |
| 90 | Cyprinidae | *Rhodeus suigensis* | 100 | EF483934 | - | - |  |
| 91 | Cyprinidae | *Rhodeus uyekii* | 100 | EF483937 | - | - |  |
| 92 | Cyprinidae | *Rhynchocypris lagowskii* | 99 | - | KJ641843 | - |  |
| 93 | Cyprinidae | *Rhynchocypris lagowskii* | 99 |  | KJ641843 |  |  |
| 94 | Cyprinidae | *Rhynchocypris lagowskii* | 99 |  | KJ641843 |  |  |
| 95 | Cyprinidae | *Rhynchocypris oxycephalus* | 99 | - | - | LC193377 |  |
| 96 | Cyprinidae | *Rhynchocypris oxycephalus* | 99 |  |  | LC193377 |  |
| 97 | Cyprinidae | *Rhynchocypris* sp. | 98 |  |  | LC193377 |  |
| 98 | Cyprinidae | *Sarcocheilichthys soldatovi* | 100 | - | - | LC146036 |  |
| 99 | Cyprinidae | *Sarcocheilichthys* sp. | 97 | KU301744 | - | AP012067 |  |
| 100 | Cyprinidae | *Sarcocheilichthys* sp. | 97 | KU301744 | - | AP012067 |  |
| 101 | Cyprinidae | *Sarcocheilichthys variegatus* | 100 | KU301744 | - | AP012067 |  |
| 102 | Siluridae | *Silurus asotus* | 100 | - | JX087351 | NC015806 |  |
| 103 | Siluridae | *Silurus microdorsalis* | 99 | KT350610 | - | - |  |
| 104 | Siluridae | *Silurus* sp. (unidentified) | 96 | KT350610 |  |  |  |
| 105 | Sinipercidae | *Siniperca scherzeri* | 100 | - | MF966985 | - | Taiwan AP014527 |
| 106 | Cyprinidae | *Squalidus chankaensis* | 100 | KT948082 | - | - |  |
| 107 | Cyprinidae | *Squalidus japonicus* | 100 |  |  | LC277782 |  |
| 108 | Cyprinidae | *Squalidus japonicus* | 99 |  |  | LC277782 |  |
| 109 | Cyprinidae | *Squalidus japonicus coreanus* | 100 | KR075134 | - |  |  |
| 110 | Cyprinidae | *Squalidus multimaculatus* | 100 | KX495606 | - | - |  |
| 111 | Bagridae | *Tachysurus fulvidraco* | 100 | - | KU133295 | LC193372 |  |
| 112 | Bagridae | *Tachysurus nitidus* | 100 | - | KC822643 | - |  |
| 113 | Cyprinidae | *Tanakia signifer* | 99 | EF483930 | - | - |  |
| 114 | Cyprinidae | *Tanakia somjinensis* | 99 | FJ515921 | - | - |  |
| 115 | Cyprinidae | *Tanakia* sp.(unidentified) | 96 | FJ515921 |  |  |  |
| 116 | Cyprinidae | *Tribolodon hakonensis* | 100 | - | - | AB626855 |  |
| 117 | Cyprinidae | *Tribolodon hakonensis* | 99 | - | - | AB626855 |  |
| 118 | Gobiidae | *Tridentiger obscurus* | 100 | KT601092 | MF663787 | LC193168 |  |
| 119 | Gobiidae | *Tridentiger radiatus* | 99 | - | EU047755 | - |  |
| 120 | Gobiidae | *Tridentiger radiatus* | 99 |  |  |  |  |
| 121 | Gobiidae | *Tridentiger trigonocephalus* | 100 | KM030481 |  |  |  |
| 122 | Gobiidae | *Tridentiger trigonocephalus* | 100 |  | KT282115 | LC385175 |  |
| 123 | Cyprinidae | *Zacco platypus* | 100 | - |  | LC277796 |  |
| 124 | Cyprinidae | *Zacco platypus* | 99 |  | KF683339 |  |  |
| 125 | Cyprinidae | *Zacco* sp. | 97 |  | KF683339 |  |  |
