# Supplementary material for: Assessment of fish biodiversity in four Korean rivers using environmental DNA metabarcoding
Source: PeerJ. 2020 Jul 14;8:e9508. doi: 10.7717/peerj.9508 (PMC7367050; doi:10.7717/peerj.9508)
Supplement: Table S2 [file peerj-08-9508-s003.docx]

Table S2. Genetic distance of species under the family Cyprinidae

|  |  | | | | | | | | | | | | | | | |
| --- | --- | --- | --- | --- | --- | --- | --- | --- | --- | --- | --- | --- | --- | --- | --- | --- |
| 1 | *Carassius auratus* (China KX505165) |  |  |  |  |  |  |  |  |  |  |  |  |  |  |  |
| 2 | *Carassius auratus* (Turkey-KM657132) | 0.006 |  |  |  |  |  |  |  |  |  |  |  |  |  |  |
| 3 | *Carassius auratus* (SJ2-99) | 0.018 | 0.012 |  |  |  |  |  |  |  |  |  |  |  |  |  |
| 4 | *Carassius auratus* (TH2-100) | 0.006 | 0.000 | 0.012 |  |  |  |  |  |  |  |  |  |  |  |  |
| 5 | *Carassius auratus* (TH3-99) | 0.006 | 0.012 | 0.012 | 0.012 |  |  |  |  |  |  |  |  |  |  |  |
| 6 | *Carassius auratus* (HS1-100) | 0.000 | 0.006 | 0.018 | 0.006 | 0.006 |  |  |  |  |  |  |  |  |  |  |
| 7 | *Carassius cuvieri* (Japan-AP011237) | 0.018 | 0.012 | 0.012 | 0.012 | 0.012 | 0.018 |  |  |  |  |  |  |  |  |  |
| 8 | *Carassius cuvieri* (SJ3-99) | 0.024 | 0.018 | 0.018 | 0.018 | 0.018 | 0.024 | 0.006 |  |  |  |  |  |  |  |  |
| 9 | *Carassius cuvieri* (TH3-100) | 0.018 | 0.012 | 0.012 | 0.012 | 0.012 | 0.018 | 0.000 | 0.006 |  |  |  |  |  |  |  |
| 10 | *Carassius gibelio* (China-KX505166) | 0.000 | 0.006 | 0.018 | 0.006 | 0.006 | 0.000 | 0.018 | 0.024 | 0.018 |  |  |  |  |  |  |
| 11 | *Cyprinus carpio* (China-MH202953) | 0.018 | 0.012 | 0.012 | 0.012 | 0.012 | 0.018 | 0.012 | 0.018 | 0.012 | 0.018 |  |  |  |  |  |
| 12 | *Cyprinus carpio* (HS2-100) | 0.030 | 0.024 | 0.024 | 0.024 | 0.024 | 0.030 | 0.024 | 0.030 | 0.024 | 0.030 | 0.012 |  |  |  |  |
| 13 | *Cyprinus carpio* (ND4-100) | 0.018 | 0.012 | 0.012 | 0.012 | 0.012 | 0.018 | 0.012 | 0.018 | 0.012 | 0.018 | 0.000 | 0.012 |  |  |  |
| 14 | *Cyprinus carpio* (ND3-99) | 0.030 | 0.024 | 0.024 | 0.024 | 0.024 | 0.030 | 0.024 | 0.030 | 0.024 | 0.030 | 0.012 | 0.024 | 0.012 |  |  |
| 15 | *Cyprinus megalophthalmus* (TH2-100) | 0.030 | 0.024 | 0.024 | 0.024 | 0.024 | 0.030 | 0.024 | 0.030 | 0.024 | 0.030 | 0.012 | 0.024 | 0.012 | 0.024 |  |
| 16 | *Cyprinus megalophthalmus* (China-KR869143) | 0.030 | 0.024 | 0.024 | 0.024 | 0.024 | 0.030 | 0.024 | 0.030 | 0.024 | 0.030 | 0.012 | 0.024 | 0.012 | 0.024 | 0.000 |
| 1 | *Acheilognathus intermedia* (Korea-EF483933) |  |  |  |  |  |  |  |  |  |  |  |  |  |  |  |
| 2 | *Acheilognathus intermedia* (SJ1-99) | 0.012 |  |  |  |  |  |  |  |  |  |  |  |  |  |  |
| 3 | *Acheilognathus macropterus* (Korea-EF483935) | 0.232 | 0.223 |  |  |  |  |  |  |  |  |  |  |  |  |  |
| 4 | *Acheilognathus macropterus* (HS1-99) | 0.223 | 0.214 | 0.018 |  |  |  |  |  |  |  |  |  |  |  |  |
| 5 | *Acheilognathus majusculus* (Japan-LC006056) | 0.198 | 0.198 | 0.127 | 0.119 |  |  |  |  |  |  |  |  |  |  |  |
| 6 | *Acheilognathus majusculus* (SJ1-99) | 0.198 | 0.198 | 0.119 | 0.112 | 0.012 |  |  |  |  |  |  |  |  |  |  |
| 7 | *Acheilognathus rhombeus* (Korea-KT601094) | 0.251 | 0.232 | 0.077 | 0.070 | 0.105 | 0.097 |  |  |  |  |  |  |  |  |  |
| 8 | *Acheilognathus rhombeus* (SJ2-99) | 0.233 | 0.215 | 0.070 | 0.063 | 0.084 | 0.077 | 0.018 |  |  |  |  |  |  |  |  |
| 9 | *Acheilognathus chankaensis* (Japan-AB016671) | 0.233 | 0.215 | 0.105 | 0.083 | 0.111 | 0.104 | 0.076 | 0.056 |  |  |  |  |  |  |  |
| 10 | *Acheilognathus koreensis* (Korea-NC013704) | 0.090 | 0.090 | 0.248 | 0.239 | 0.240 | 0.231 | 0.287 | 0.268 | 0.278 |  |  |  |  |  |  |
| 11 | *Acheilognathus yamatsutae* (Korea-NC013712) | 0.205 | 0.205 | 0.111 | 0.104 | 0.070 | 0.056 | 0.083 | 0.063 | 0.111 | 0.229 |  |  |  |  |  |
| 12 | *Acheilognathus signifer* (Korea-EF483930) | 0.063 | 0.063 | 0.231 | 0.222 | 0.205 | 0.197 | 0.249 | 0.232 | 0.241 | 0.024 | 0.204 |  |  |  |  |
| 13 | *Tanakia signifer* (SJ1-99) | 0.070 | 0.070 | 0.240 | 0.231 | 0.214 | 0.205 | 0.259 | 0.241 | 0.251 | 0.018 | 0.213 | 0.006 |  |  |  |
| 14 | *Acheilognathus somjinensis* (Korea-FJ515921) | 0.084 | 0.070 | 0.223 | 0.214 | 0.206 | 0.198 | 0.232 | 0.215 | 0.215 | 0.056 | 0.205 | 0.031 | 0.037 |  |  |
| 15 | *Tanakia somjinensis* (SJ2-99) | 0.091 | 0.077 | 0.232 | 0.223 | 0.215 | 0.206 | 0.241 | 0.224 | 0.224 | 0.063 | 0.214 | 0.037 | 0.044 | 0.006 |  |
| 1 | *Nipponocypris koreanus* (China-KJ427719) |  |  |  |  |  |  |  |  |  |  |  |  |  |  |  |
| 2 | *Nipponocypris koreanus* (TH1-100) | 0.000 |  |  |  |  |  |  |  |  |  |  |  |  |  |  |
| 3 | *Nipponocypris temminckii* (Japan-LC468890) | 0.011 | 0.011 |  |  |  |  |  |  |  |  |  |  |  |  |  |
| 4 | *Nipponocypris temminckii* (HS1-100) | 0.011 | 0.011 | 0.000 |  |  |  |  |  |  |  |  |  |  |  |  |
| 1 | *Rhodeus uyekii* (Korea-EF483937) |  |  |  |  |  |  |  |  |  |  |  |  |  |  |  |
| 2 | *Rhodeus suigensis (*Korea-EF483934) | 0.164 |  |  |  |  |  |  |  |  |  |  |  |  |  |  |
| 3 | *Rhodeus suigensis* (SJ2-100) | 0.164 | 0.000 |  |  |  |  |  |  |  |  |  |  |  |  |  |
| 4 | Rhodeus uyekii (SJ1-100) | 0.000 | 0.164 | 0.164 |  |  |  |  |  |  |  |  |  |  |  |  |
