# Supplementary material for: Assessment of fish biodiversity in four Korean rivers using environmental DNA metabarcoding
Source: PeerJ. 2020 Jul 14;8:e9508. doi: 10.7717/peerj.9508 (PMC7367050; doi:10.7717/peerj.9508)
Supplement: Table S3 [file peerj-08-9508-s004.docx]

Table S3. List of the invasive fish species identified from four river eDNA metabarcoding study

| No. | River | Species Name | Family | Order | Identity (%) |
| --- | --- | --- | --- | --- | --- |
| 1 | Hyeongsan | *Carassius cuvieri* | Cyprinidae | Cypriniformes | 100.0 |
| 2 |  | *Cyprinus carpio* | Cyprinidae | Cypriniformes | 100.0 |
| 3 |  | *Cyprinus megalophthalmus* | Cyprinidae | Cypriniformes | 100.0 |
| 4 |  | *Micropterus salmoides* | Centrarchidae | Centrarchiformes | 100.0 |
| 5 |  | *Lepomis macrochirus* | Centrarchidae | Centrarchiformes | 100.0 |
| 6 | Nakdong | *Cyprinus carpio* | Cyprinidae | Cypriniformes | 100.0 |
| 7 |  | *Cyprinus megalophthalmus* | Cyprinidae | Cypriniformes | 100.0 |
| 8 |  | *Micropterus salmoides* | Centrarchidae | Centrarchiformes | 100.0 |
| 9 |  | *Lepomis macrochirus* | Centrarchidae | Centrarchiformes | 100.0 |
| 10 | Seomjin | *Carassius cuvieri* | Cyprinidae | Cypriniformes | 99.42 |
| 11 |  | *Micropterus salmoides* | Centrarchidae | Centrarchiformes | 99.41 |
| 12 | Taehwa | *Carassius cuvieri* | Cyprinidae | Cypriniformes | 100.0 |
| 13 |  | *Cyprinus carpio* | Cyprinidae | Cypriniformes | 100.0 |
| 14 |  | *Cyprinus megalophthalmus* | Cyprinidae | Cypriniformes | 100.0 |
| 15 |  | *Micropterus salmoides* | Centrarchidae | Centrarchiformes | 100.0 |
| 16 |  | *Lepomis macrochirus* | Centrarchidae | Centrarchiformes | 100.0 |
